# Alumina-Supported NiMo Hydrotreating Catalysts—Aspects of 3D Structure, Synthesis, and Activity

**DOI:** 10.1021/acs.jpcc.2c05927

**Published:** 2022-10-24

**Authors:** Mengyan Li, Johannes Ihli, Marcel A. Verheijen, Mirko Holler, Manuel Guizar-Sicairos, Jeroen A. van Bokhoven, Emiel J. M. Hensen, Thomas Weber

**Affiliations:** † Laboratory of Inorganic Materials and Catalysis, Department of Chemical Engineering and Chemistry, Eindhoven University of Technology, Het Kranenveld 14, 5600 MBEindhoven, The Netherlands; ‡ Paul Scherrer Institute, 5232Villigen PSI, Switzerland; § Department of Applied Physics, Eindhoven University of Technology, 5600 MBEindhoven, The Netherlands; ∥ Eurofins Materials Science, 5656 AEEindhoven, The Netherlands; ⊥ Institute for Chemical and Bioengineering, Department of Chemistry and Applied Biosciences, ETH Zurich, Vladimir-Prelog-Weg 1-5/10, 8093Zurich, Switzerland

## Abstract

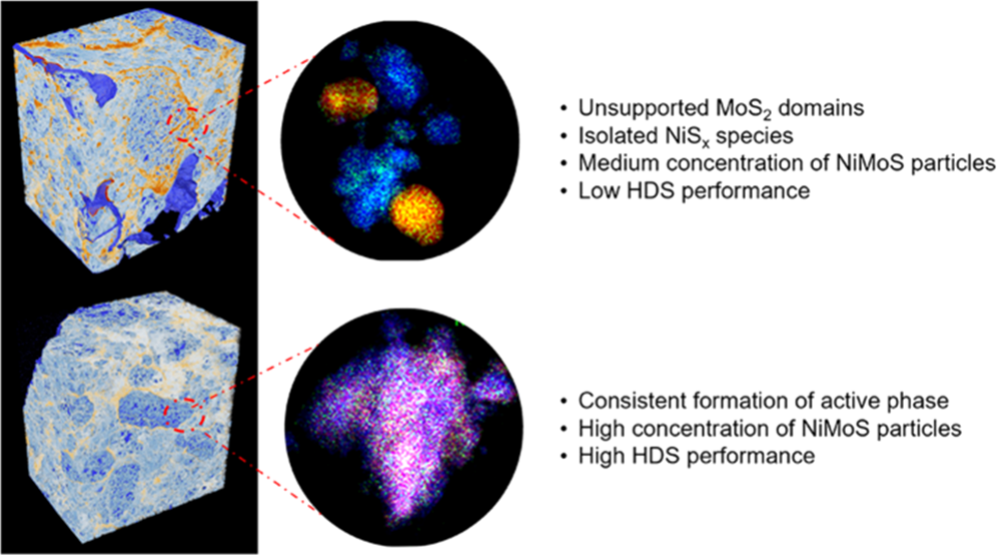

Preparation conditions have a vital effect on the structure of alumina-supported hydrodesulfurization (HDS) catalysts. To explore this effect, we prepared two NiMoS/Al_2_O_3_ catalyst samples with the same target composition using different chemical sources and characterizing the oxidic NiMo precursors and sulfided and spent catalysts to understand the influence of catalyst structure on performance. The sample prepared from ammonium heptamolybdate and nickel nitrate (sample A) contains Mo in the oxidic precursor predominantly in tetrahedral coordination in the form of crystalline domains, which show low reducibility and strong metal–support interactions. This property influences the sulfidation process such that the sulfidation processes of Ni and Mo occur tendentially separately with a decreased efficiency to form active Ni–Mo–S particles. Moreover, inactive unsupported MoS_2_ particles or isolated NiS*_x_* species are formed, which are either washed off during catalytic reaction or aggregated to larger particles as seen in scanning transmission electron microscopy/energy-dispersive X-ray spectroscopy (STEM/EDX). The oxidic precursor of the sample synthesized using nickel carbonate and molybdenum trioxide as metal sources (sample B), however, contains Mo in octahedral coordination and shows higher reducibility of the metal species as well as weaker metal–support interactions than that of sample A; these properties allow an efficient sulfidation of Mo and Ni such that formation of active Ni–Mo–S particles is the main product. Ptychographic X-ray computed tomography (PXCT) and STEM and EDX measurements show that the structure formed during sulfidation is stable under operation conditions. The structural differences explain the HDS activity difference between these two samples and explain why sample B is much active than sample A.

## 1 Introduction

Catalytic hydrodesulfurization (HDS) is a mature technology used to remove sulfur from crude oil to produce ultraclean fuels. Typically, HDS catalysts consist of sulfides of Mo and Ni or Mo and Co, supported on a high-surface-area alumina (γ-Al_2_O_3_)^1^ carrier. These catalysts are usually prepared in the form of oxidic precursors by impregnating the carrier with the metals followed by drying and calcination. Catalyst activation is done by sulfidation in a H_2_S/H_2_ gas flow at elevated temperatures (around 400 °C), yielding NiMo or CoMo sulfide phases. The structure of these sulfide phases is described in the so-called Co–Mo–S model;^2^ Co or Ni promotor atoms are located at the edges of nanosized MoS_2_ particles. With sharpening global environmental regulations, a strong demand for fuels containing only traces of sulfur (10 ppm or less) was created. To satisfy market needs, highly active catalysts of increased lifetime and stability are needed, prompting the exploration of several synthesis processes.^3,4^

The traditional workflow of producing HDS catalysts consists of impregnation, drying, calcination, and activation (sulfidation). In the early 2000s, a modified workflow gained traction since it yielded catalysts of higher activity. In this workflow, organic additives, such as glycols, ethylenediaminetetraacetic acid (EDTA), or nitrilotriacetic acid (NTA), were added to the catalyst formulation.^5−8^ Since these additives would decompose or oxidize under typical calcination conditions (400 °C in air), the calcination step was omitted. The effect of organic additives on catalytic performance has been explored in numerous studies, linking changes in structure, density, and stability of the active phase to performance.^6,9−21^ These uncalcined catalyst types show a low tendency to form Mo–O–Al bonds, which advances the formation of type II catalysts. Compared to the organic additive-free and calcined type I catalysts, which are characterized by a strong interaction with the support, type II catalysts are generally more active.^22^ Besides organic additives, phosphorus is another important component in the formation of highly active hydrotreating catalysts;^23,24^ experimental observations have shown that the presence of phosphorus promotes the stacking of MoS_2_-type particles by lowering metal–support interactions and enhancing the formation of type II phases.^25−27^

A critical variable in catalyst preparation is the metal formulation. Since the series of oxomolybdates with the monomeric MoO_4_^2–^ anion and the neutral solid MoO_3_ as end members are accessible via pH-dependent aggregation processes,^28,29^ there are several ways to design the impregnation solution. The most commonly used recipe to prepare Ni-, Mo-, and P-containing hydrotreating catalysts utilizes aqueous solutions of ammonium heptamolybdate ((NH_4_)_6_Mo_7_O_24_), nickel nitrate (Ni(NO_3_)_2_), and phosphoric acid.^25,30^ Moving toward the end members of the oxymolybdate series, molybdenum trioxide (MoO_3_), nickel carbonate (NiCO_3_) and phosphoric acid,^31^ or sodium molybdate (Na_2_MoO_4_·2H_2_O) and nickel nitrate (Ni(NO_3_)_2_)^32^ are other feasible sources of Mo and Ni. Some studies utilized highly condensed and symmetric starting materials such as Keggin^33,34^ and Anderson complexes.^35,36^ Overall, there is no clear answer as to which type of impregnation solution yields the best catalysts.

Characterization studies conducted over the years have a focus on describing the active NiMoS phase at atomic or nanometer scale, e.g., utilizing EXAFS to explore the coordination number of Mo^37−40^ to determine particle size and staking degree of NiMoS particles, or the degree of edge decoration through promotor atoms. Active site(s) and molecular reaction routes of Co–Mo–S structures synthesized on Au(111) were monitored by means of scanning tunneling microscopy (STM),^41^ and first-principles calculations were used to describe particle structure^42,43^ as well as mechanisms of reactions taking place at the active sites located on the edges of Co–Mo–S and Ni–Mo–S nanocrystals.^42,44,45^ To a much lesser extent, information regarding metal dispersion on alumina or the influence and change of the alumina pore structure on and during metal deposition can be found.

Here, we investigate two hydrotreating catalysts, where malic acid (MA) and phosphorus were added to the NiMo catalyst formulation by co-impregnation.^6,7^ These two catalysts were prepared on the same alumina carrier with identical metal and phosphorus loadings and contain the same organic additive. The only difference is that they were prepared using two different impregnation solutions. Catalyst A was prepared with (NH_4_)_6_Mo_7_O_24_ and Ni(NO_3_)_2_ as Mo and Ni sources (route A), while catalyst B was prepared from MoO_3_ and NiCO_3_ (route B). Thus, the difference lies in the impregnation chemistry brought about by these impregnation solutions, which will be shown to lead to substantial structural differences of the active phases. For a more comprehensive understanding of the structure difference, we present a detailed structural characterization of these two samples and discuss their performance levels on structural grounds. Catalyst characterization is based on physical methods, spectroscopy, diffraction, tomography, and catalytic activity measurements. In particular, we used N_2_ physisorption and temperature-programmed reduction (TPR) as physical methods, and X-ray photoelectron spectroscopy (XPS), Raman, and X-ray diffraction (XRD) as standard spectroscopy and diffraction methods. 2D and 3D imaging methods including scanning transmission electron microscopy (STEM), energy-dispersive X-ray spectroscopy (EDX), and ptychographic X-ray computed tomography (PXCT) were used for advanced structural characterization. The catalytic activity of our samples was evaluated in thiophene, dibenzothiophene, and gas oil hydrodesulfurization tests.

## 2 Experimental Section

### 2.1 Materials

Ammonium heptamolybdate tetrahydrate ((NH_4_)_6_Mo_7_O_24_·4H_2_O, 99%), nickel carbonate (NiCO_3_, 48–50%), phosphoric acid (H_3_PO_4_, 85 wt % in H_2_O), dl-malic acid (C_4_H_6_O_5_, 98%), thiophene (C_4_H_4_S, 99%), adamantane (C_10_H_16_, 99%), and dibenzothiophene (C_12_H_8_S, 98%) were purchased from Sigma-Aldrich. Molybdenum trioxide (MoO_3_, 99.9%) was purchased from Climax. Nickel nitrate hexahydrate (Ni(NO_3_)_2_·6H_2_O, 98%), ammonium hydroxide (NH_3_·H_2_O, 28%), and n-hexadecane (CH_3_(CH_2_)_14_CH_3_, 99%) were purchased from Alfa Aesar. All chemicals were used without further purification. We used the same industrial γ-Al_2_O_3_ carrier (BET surface area: 300 m^2^ g^–1^; pore volume: 0.82 cm^3^ g^–1^; average pore diameter: 8.5 nm) as in previous studies.^46,47^

### 2.2 Catalyst Preparation

Two types of NiMo/Al_2_O_3_ catalysts prepared from two sets of different metal salts with malic acid (MA) as additive were prepared by incipient wetness impregnation of γ-Al_2_O_3_^48^ extrudates with an aqueous solution of respective metal salts and additive. The target composition of both types is 15 wt % Mo, 3.67 wt % Ni, 2 wt % P (referenced to MoO_3_, NiO, and P_2_O_5_), and MA in a molar ratio of 1.2 per atom Mo. Phosphorus was added in the form of H_3_PO_4_, which does also facilitate metal dissolution.^24,49^ The two types of NiMo/Al_2_O_3_ catalysts and associated preparation routes are subsequently referred to as sample/route A and B.

*Route A.* (NH_4_)_6_Mo_7_O_24_·4H_2_O (8.1 g) was slowly added to an aqueous solution of H_3_PO_4_ (2.2 g, 85 wt % in H_2_O). Ammonia (28%) was added following yielding an initially clear solution, which under continuous stirring results in a light-yellow solution. Next, 5.3 g of Ni(NO_3_)_2_·6H_2_O was added under stirring, which results in a transparent green solution. To obtain the final impregnation solution, 7.4 g of MA was added. By adding water, the volume of the impregnation solution was finally adjusted to the pore volume of alumina. The pH of the final impregnation solution is 5.

*Route B.* MoO_3_ (6.6 g), NiCO_3_ (2.2 g), and H_3_PO_4_ solution (2.2 g, 85 wt % in H_2_O) were dissolved in deionized water under constant stirring and heated to 100 °C yielding a clear green solution to which 7.4 g of MA were added. The pH of the final solution is <1.

Impregnation solution (16.4 mL) was then added to each 20 g of alumina extrudes (diameter: 1 mm, length: 3–13 mm). The extrudates were kept for a period of 2 h under slow movement on a roller bank and subsequently dried overnight at 120 °C yielding the catalyst precursors (oxide). To preserve the malic acid for the subsequent sulfidation process, the prepared samples were not calcined.^50^ Freshly sulfided catalysts (A and B) and spent samples recovered from the gas oil HDS test are referred to as (sulfide) and (spent), respectively. The final metal loading of these catalysts was determined by inductively coupled plasma optical emission spectroscopy (ICP-OES), and the results are compiled in Table 1.

**Table 1:** Catalyst Composition, Surface Area, Pore Volume, and Diameter

	loading (wt %)^a^						
catalyst	Mo	Ni	P	Ni/Mo molar ratio^a^	surface area^b^(m^2^/g)	pore volume^b^,^c^(cm^3^/g)	pore diameter^b^,^c^ (nm)
A (oxide)	11.32	2.65	1.53	0.4	112	0.2	10.4
A (sulfide)	10.42	2.81	1.44	0.4			
A (spent)	9.16	2.57	1.33	0.5			
B (oxide)	11.36	2.70	1.57	0.4	201	0.2	7.2
B (sulfide)	10.70	2.56	1.43	0.4			
B (spent)	10.69	2.57	1.31	0.4			
γ-Al_2_O_3_					300	0.8	9.0

a Determined by ICP-AES.

b Determined by N_2_ physisorption.

c Values determined from the BJH method applied to the adsorption branch of the isotherm (Figure S1).

### 2.3 Catalyst Activation

To prepare the sulfided catalyst samples, ∼50 mg of oxidic precursor (75–125 μm) were loaded in the middle of a valve-sealed stainless-steel reactor with an inner diameter of 4 mm and heated up to 350 °C with a 2 °C/min ramp rate in 1 bar H_2_/H_2_S (10% v/v) at a flow rate of 50 mL/min. The sample was kept under these conditions for 2 h and subsequently cooled to room temperature in He atmosphere. The sulfided catalyst samples were then transferred to and stored in a N_2_-filled glovebox for further characterization. During sulfidation, malic acid is decomposed and the decomposition products are released. The final active catalyst does not contain any organic compounds.

### 2.4 Catalytic Activity Measurements

#### 2.4.1 Thiophene HDS Activity

To avoid contact with air, catalyst activation and activity tests were performed sequentially in a stainless-steel reactor with an inner diameter of 4 mm at ambient pressure. In detail, ∼76 mg of oxidic precursor (75–125 μm) was mixed with 200 mg of SiC and loaded in the middle of the reactor. The samples were then sulfided under a constant flow of H_2_/H_2_S (10% v/v) at a flow rate of 50 mL/min at 350 °C for 2 h. The temperature was then increased to 400 °C and the inflow switched to a reaction or testing feed composed of 4% (v/v) thiophene in H_2_ (100 mL/min) for 13 h. The steady-state catalyst activity was measured by gas chromatography (GC) equipped with a flame ionization detector (FID) and an RTX-1 column with 0.32 mm ID. The normalized reaction rate (*r*_Thio_) was calculated according to
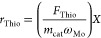
1where *F*_Thio_ is the molar flow of thiophene in mol_Thio_ h^–1^, *m*_cat_ is the catalyst mass in g, ω_Mo_ is the fraction of metal in mol_Mo_ g_cat_^–1^, and *X* is the conversion.

#### 2.4.2 Dibenzothiophene (DBT) HDS Activity

DBT HDS activity was measured in a fixed-bed high-pressure reactor under gas and liquid feed trickle flow conditions. The reactor (I.D.: 4 mm) was packed with 200 mg of oxidic precursor (75–125 μm) diluted with 1 g of SiC. To activate the catalyst, we heated the reactor to 350 °C at a heating rate of 2 °C/min in a 50 mL/min H_2_/H_2_S (10% v/v) and kept there for 2 h. After that, the oven temperature was adjusted to 270 °C, the pressure was increased to 20 bar, and the feed was switched to the reaction feed containing 4 wt % DBT and 2 wt % adamantane in n-hexadecane, with a liquid hourly space velocity (LHSV) of 9.2 h^–1^ and a H_2_/feed ratio of 200 L kg ^–1^. Adamantane is used as the internal reference compound for GC analysis. The steady-state activity was determined after 12 h reaction. Products were analyzed by online GC-FID equipped with RTX-1 column with 0.32 mm I.D. and 30 m in length. The reaction rate constant was calculated via
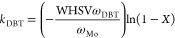
2where WHSV is the weight hourly space velocity, ω_DBT_ is the fraction of DBT, ω_Mo_ is the fraction of Mo and *X* is the conversion.

#### 2.4.3 Gas-Oil HDS Activity

Gas-oil activity tests were conducted in a fixed-bed high-pressure reactor with a trickle flow of gas and liquid feed (60 bar, H_2_/feed = 350 NL/kg, liquid hourly space velocity (LHSV): 1.2 h^–1^). The reactor, 4 mm ID, was packed with 0.75 mL of catalyst extrudates and sandwiched between two 10 cm layers of Zirblast. Before reaction, the catalysts were pretreated with a sulfidation feed (gas oil feed spiked with 2.69 wt % Sulfurzol) at 200 °C for 5 h, followed by heating to 280 °C for 5 h and finally to 315 °C for 5 h. Afterward, the temperature was lowered to 200 °C, the sulfidation feed switched to the testing gas oil feed (1.28 wt % S, 234 ppm N) and the temperature subsequently increased to the target run temperature, under which the S content in the product stream is ≤10 ppm. The desired run temperature was analyzed 470 h after the start of the test. The sulfur content in the products was analyzed by atomic emission spectroscopy-inductively coupled plasma-mass spectroscopy (AES-ICP-MS). After the run, the samples were extracted from the reactors in a nitrogen-operated glovebox and stored in sealed vessels for further characterization.

### 2.5 Catalyst Characterization

#### 2.5.1 N_2_ Physisorption

Textural properties, i.e., specific surface area, pore volume, and average pore size, were determined by means of N_2_ physisorption in a Micromeritics AutoChem apparatus. Prior to physisorption measurements, samples were pretreated overnight with N_2_ at 120 °C.

#### 2.5.2 XRD

Crystalline phases were determined with a Bruker D2 Phase powder diffraction system using Cu Kα radiation. The acquired X-ray diffraction (XRD) patterns covered an angular range of 2θ = 10–70°. The patterns were recorded with a step size of 0.01° and a step acquisition time of 0.5 s. The average crystallite size, *L*, was calculated using the Scherrer equation
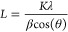
3where *K* is a dimensionless shape factor, with a value close to unity; λ is the wavelength of the X-ray radiation; β is the line broadening at half the maximum intensity (FWHM), and θ is the Bragg angle. Instrument broadening was taken into account.

#### 2.5.3 TPR

TPR measurements were performed using a Micromeritics AutoChem II 2920 equipped with a fixed-bed reactor and a thermal conductivity detector (TCD). Around 50 mg of the respective sample materials were loaded into a glass reactor and pretreated at 200 °C for 1 h in a 50 mL/min helium flow. The temperature was then increased to 900 °C at 5 °C/min in a H_2_/He (5% v/v) mixture and kept for 1 h, while the hydrogen consumption was monitored by TCD.

#### 2.5.4 Raman Spectroscopy

Raman spectra were acquired using a confocal Witec α 300 R microscope equipped with a 532 nm diode excitation source, a 1200 lines/mm grating (BLZ = 500 nm), and a CCD detector. A Zeiss LD EC Epiplan-Neofluar Dic 50×/0.55 objective was used. The presented spectra are the result of 30 accumulations with an acquisition time of 10 s per accumulation.

#### 2.5.5 XPS

XPS spectra were recorded at room temperature using a Thermo Scientific K-Alpha spectrometer, equipped with a monochromatic small-spot X-ray source (Al Kα = 1486.6 eV) operating at 72 W and a spot size of 400 μm. Sulfided and spent samples were prepared in a N_2_-operated glovebox. The mechanically ground samples were dispersed on a carbon tape-covered alumina holder, which was then transferred into the XPS apparatus via an airtight transport vessel. The whole process was done without exposing the sample to air. For the measurement, the background pressure was 2 × 10^–9^ mbar. The survey scan and region scan were measured using a constant pass energy of 160 and 40 eV respectively. Data analysis was performed using the CasaXPS software with a Shirley background subtraction and Gaussian–Lorentzian fitting procedure, where the binding energy (B.E.) was calibrated using the C 1s peak at 284.8 eV as reference.

#### 2.5.6 PXCT

3D structural measurements were carried out by means of PXCT.^47,51,52^ PXCT is a lensless quantitative imaging technique in which each tomographic projection is calculated by means of ptychographic phase-retrieval algorithms.^53,54^ Tomographic reconstruction retrieves the complex-valued refractive index of the examined sample, providing tomograms of both phase and amplitude contrast.^54^ Away from sample-relevant absorption edges, the retrieved refractive index decrement values can be converted to electron density as described in Diaz et al.^55^ Measurements were carried out at the cSAXS beamline of the Swiss Light Source at 6.2 keV photon energy at room temperature in an inert atmosphere. A series of sample cylinders, ≈25 μm in diameter, extracted central, from a catalyst pellet of samples A (sulfide), B (sulfide), A (spent), and B (spent) were examined. The sample cylinders or pillars were prepared using a micro-lath and focus-ion-beam (FIB) milling (Figure S2).^56^ The obtained quantitative electron density tomograms possess, on average, a half-period spatial resolution of 40 nm. The resolution was evaluated through Fourier shell correlation (FSC) (Figure S3).^57^ Details regarding tomogram acquisition and analysis can be found in the Supporting Information.

#### 2.5.7 STEM and EDX

The local morphology and elemental composition of the sulfide and spent catalysts were determined by STEM and EDX mapping using a probe-corrected JEOL ARM 200F transmission electron microscope operating at an acceleration voltage of 200 kV. The preparation of the sulfide and spent samples was conducted in a glovebox, specifically, around 5 mg of catalyst sample were dispersed in n-hexane to make a suspension, a few droplets of which was then placed on a Cu grid. The grid was then transported to STEM. The mean length of individual MoS_2_ platelets and the average number of layers per particle were calculated from acquired STEM images using ImageJ. The mean length was determined by fitting a log-normal function to the platelets size distribution. The degree of stacking (*N*) was calculated according to eq 4.
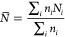
4where *N*_*i*_ is the number of MoS_2_ layers within a particle and *n*_*i*_ is the amount of individual MoS_2_ platelets counted for a given number of layers *N*_*i*_.

The local elemental distribution in the sulfide and spent catalysts was determined by STEM-EDX mapping using the same JEOL ARM 200F by means of a 100 mm^2^ (1 srad) Centurio SDD EDX detector. The correlation between Mo and Ni was calculated via MATLAB, as shown in Figure S8, detailed information can be found in the Supporting Information.

## 3 Results

### 3.1 Catalytic Activity

Table 2 shows the catalytic activity of samples A and B in gas-phase thiophene HDS tests at atmospheric pressure, liquid-phase DBT HDS at 20 bar, and liquid-phase gas oil HDS at 60 bar. The catalytic activity of sample B in atmospheric thiophene HDS is higher than that of sample A, i.e., 101.9 mol mol_Mo_^–1^ h^–1^ compared to 53.4 mol mol_Mo_^–1^ h^–1^. In DBT HDS at 270 °C, the activity difference is significantly larger as can be derived from respective reaction rate constants of 17.7 and 81.2 mol mol_Mo_^–1^ h^–1^ for samples A and B, respectively. The gas oil HDS test also clearly differentiates the two samples, i.e., 4.96 mol mol_Mo_^–1^ h^–1^ for sample A and 22.31 mol mol_Mo_^–1^ h^–1^ for sample B. The products of the DBT HDS reaction are also listed in Table 2, where it can be noted that the main products are biphenyl (BP) and cyclohexylbenzene (CHB), no bicyclohexyl (BCH) was observed under our reaction conditions.

**Table 2:** Gas-Phase Thiophene HDS Reaction Rates and Liquid-Phase Dibenzothiophene HDS Reaction Rate Constants of A (Sulfide) and B (Sulfide)

	thiophene		DBT					gas-oil
					selectivity			
catalyst	reaction rates (mol mol_Mo_^–1^ h^–1^)	conversion (%)	reaction temperature (°C)	conversion (%)	BP	CHB	reaction rate constants (mol mol_Mo_^–1^ h^–1^)	reaction rate constants (mol mol_Mo_^–1^ h^–1^)
A (sulfide)	53.4 ± 0.8	46.1	230	7.6	90.0	10.0	3.4	4.96
			250	16.7	83.8	16.2	7.8	
			270	34.1	75.6	24.4	17.7	
B (sulfide)	101.9 ± 0.8	87.2	230	25.4	57.8	42.2	12.5	22.31
			250	51.3	54.3	45.7	30.6	
			270	85.2	50.7	49.3	81.2	

### 3.2 Physiochemical Bulk Characterization of the Oxidic Catalyst Precursor

**Figure 1:**
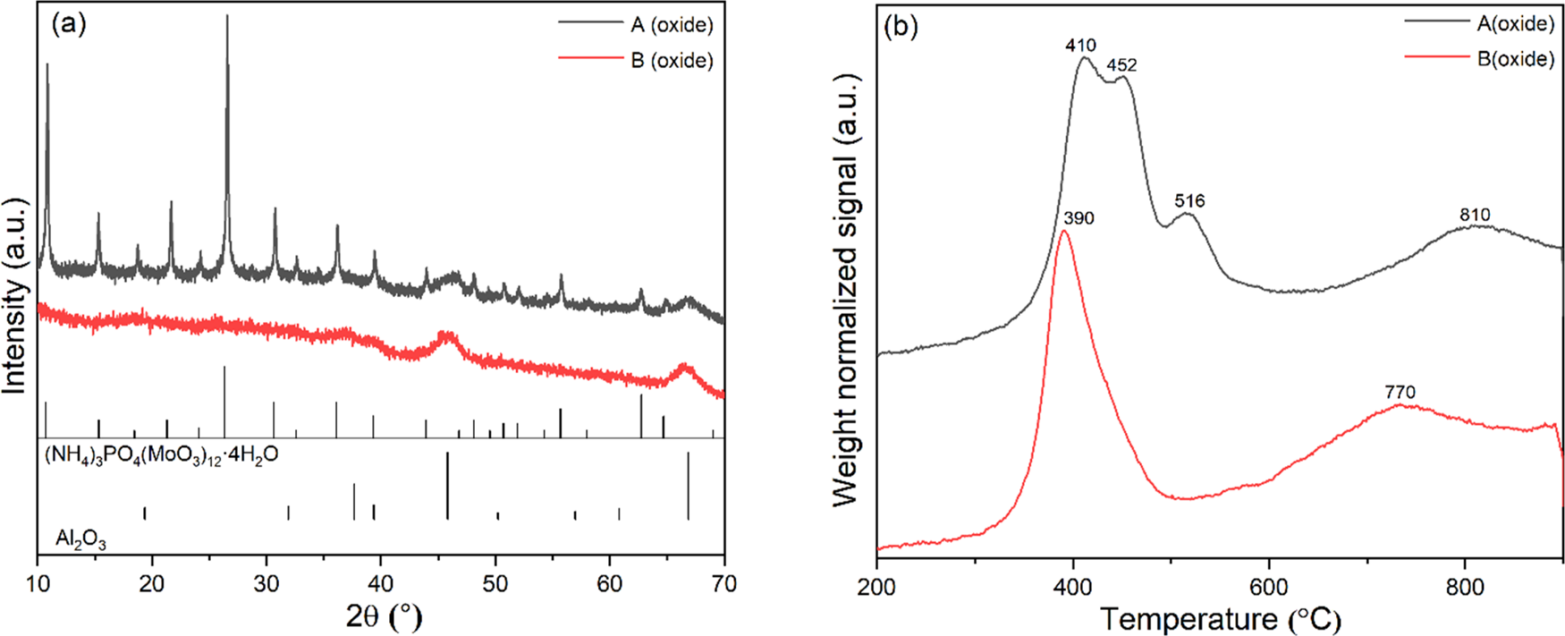
(a) XRD pattern and (b) TPR curves of samples A (oxide) and B (oxide).

**Figure 2:**
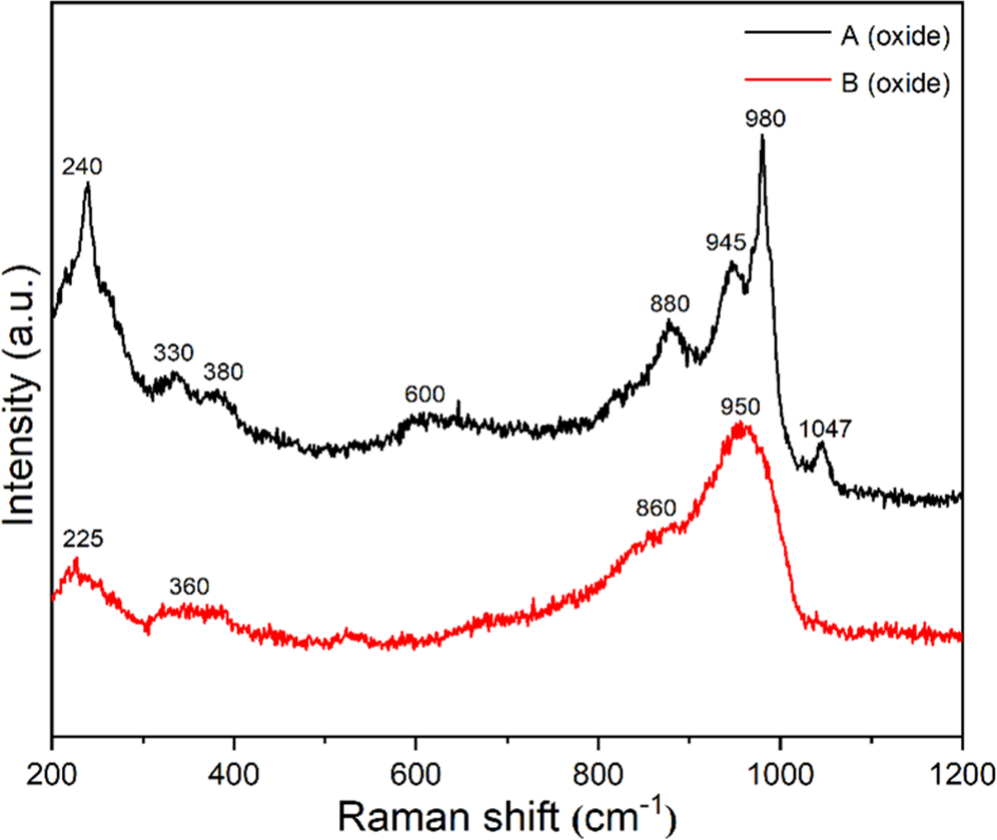
Raman spectra of samples A (oxide) and B (oxide).

To investigate the origin of these activity differences we first acquired powder XRD (Figure 1a), TPR (Figure 1b), BET data (Table 1 and Figure S1), and Raman spectra (Figure 2) of the supported catalysts in their oxidic precursor form.

#### 3.2.1 XRD

Sample B (oxide) exhibits characteristic diffraction peaks at 2θ = 46 and 67° attributed to γ-Al_2_O_3_ (JCPDS 01-077-0396). The absence of other diffraction features points to highly dispersed, nanoscopic or amorphous species. The XRD pattern of sample A (oxide) is different. Next to the above-mentioned reflections caused by γ-Al_2_O_3_, a set of sharp diffraction peaks at 2θ = 10.7, 15.3, 18.4, 21.2, 24.0, 26.3, 30.6, 32.5, 36.0, 43.9, 48.1, 50.6, 51.9, 55.6, 62.7, and 64.7° indicate the presence of crystalline (NH_4_)_3_PO_4_(MoO_3_)_12_·4H_2_O (JCPDS 00-009-0412). The crystallite size as estimated from the Scherrer equation^58^ is 60 nm. (NH_4_)_3_PO_4_(MoO_3_)_12_·4H_2_O crystallites may form in the impregnation solution or on the alumina surface upon drying. The differences in the metal speciation on the alumina surface in the two samples lead to a higher surface area for sample B (oxide) and account for the different N_2_ physisorption results (Table 1).

The reducibility of samples A (oxide) and B (oxide) was explored by means of TPR. Overall, their reduction profiles are rather similar. In Figure 1b, both catalyst samples exhibit dominant reduction peaks around 400 and 800 °C. The low-temperature reduction peaks can be attributed to a reduction of Ni oxide species, as well as a partial reduction of polymolybdates that have a weak interaction with the support (Mo^6+^ to Mo^4+^).^59,60^ The broad peak at the higher temperature is attributed to the deep reduction of all Mo species, including tetrahedrally coordinated Mo^4+^ species. The differences in TPR profiles between samples are as follows. In the low-temperature reduction region, sample A (oxide) shows three reduction peaks at 410, 452, and 516 °C, while only one peak occurs at 390 °C in sample B (oxide). Differences in Mo–support interactions due to distinctly different oxomolybdate species likely account for the existence of three reduction peaks in the low-temperature reduction area in A (oxide). In sample B (oxide), differences in the precursor structures are small, so that Mo–support interactions are more uniform and result in one (broad) reduction peak. Consistently, the high-temperature reduction peak in sample B (oxide) is centered around 770 °C and is ∼40 °C lower than the corresponding peak in A (oxide), again indicating a weaker metal–support interaction in B (oxide). This type of interaction for sample B also leads to higher MoS_2_ crystallites stacking upon sulfidation and is commonly agreed as a requirement for the formation of type II Ni–Mo–S phases.^25,61,62^

Following, we used Raman spectroscopy to determine the nature of Mo and Ni oxide species present on the alumina surface (Figure 2).^11^ The Raman spectra of sample B (oxide) show a broad band at 950 cm^–1^ together with a shoulder at 860 cm^–1^ and two less intense bands at 360 and 225 cm^–1^, which are considered to be the vibrational signature of octahedrally coordinated polymolybdate species.^63−65^ The spectrum of sample A (oxide), exhibits in addition a band at 1047 cm^–1^, which is assigned to the ν_s_(NO_3_) stretching vibration of nitrate anions (originating from the nickel nitrate source).^66^ The main bands at 980, 880, 600, 380, and 240 cm^–1^ are due to [PMo_12_O_40_]^3–^ species. Detailed band assignments can be found in refs (67, 68). In addition, to the band position implied differences in coordination state, the shape of the bands themselves is worth noting. The widths of Raman bands are frequently positively correlated with the degree of crystallinity of the probed material, as such the sharp bands in the spectrum of sample A (oxide) point to mainly crystalline species, while the broad bands in the spectrum of sample B (oxide) indicate a higher degree of structural disorder. The Mo coordination difference observed above already exists in the respective impregnation solutions (Figure S4), and the reason for this difference is that Mo aggregation is a pH-dependent process yielding different oxomolybdates, e.g., MoO_4_^2–^ and MoO_3_, as explained in Section 1.

### 3.3 Changes in Catalyst Composition during Activation/Sulfidation

**Figure 3:**
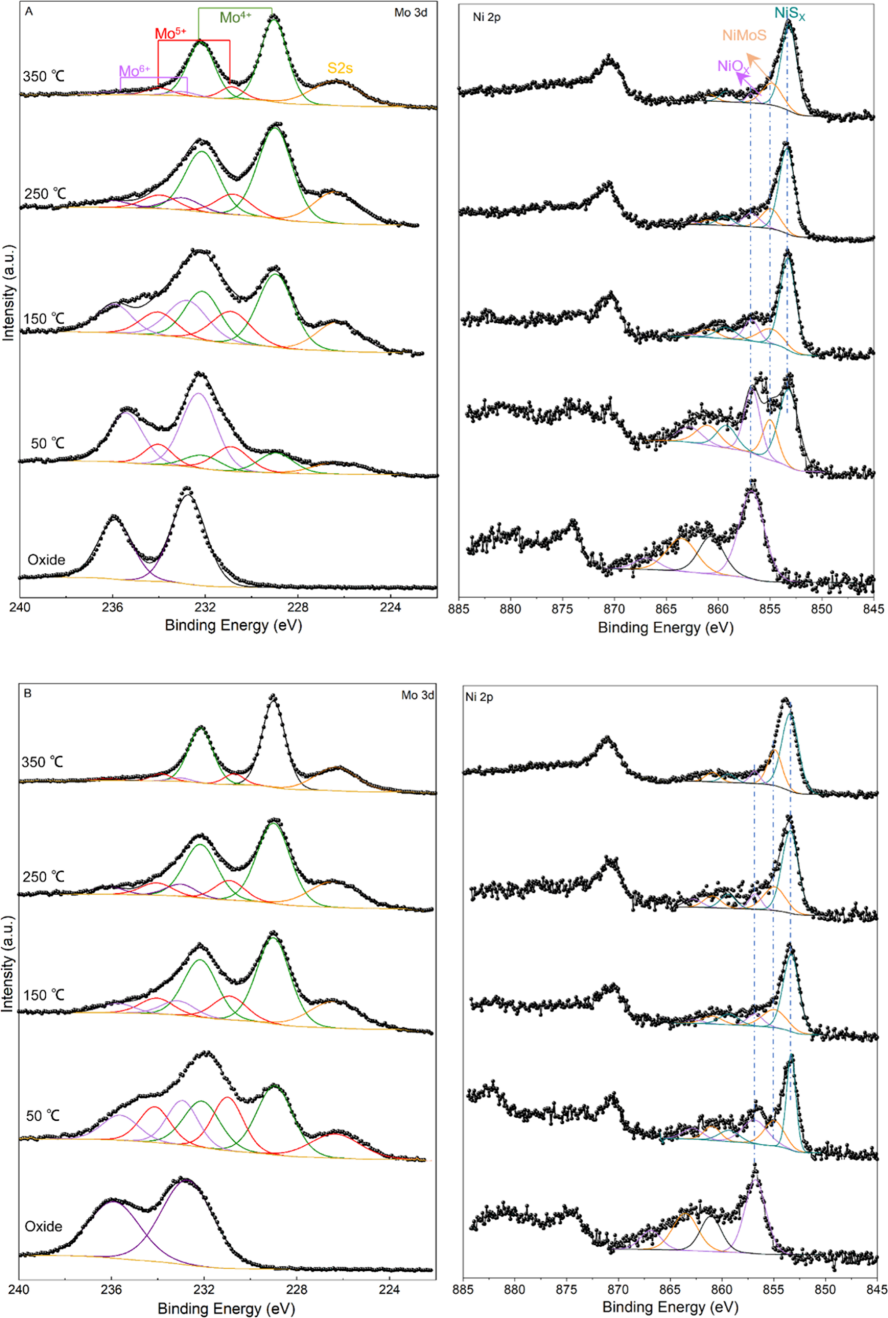
Mo 3d and Ni 2p XPS spectra of samples A (top) and B (bottom) obtained after sulfidation (room temperature to 350 °C, 1 bar H_2_S/H_2_). Experimental data are presented by open circles and the fit by a black curve. The fits for Mo 3d are composed of Mo^4+^ (green), Mo^5+^ (pink), Mo^6+^ (purple), as well as S^2–^ 2s signal (yellow). Ni 2p_3/2_ fits are composed of Ni as in NiO*_x_* (purple), NiMoS (orange), and NiS*_x_* (blue).

Temperature-dependent changes during sulfidation of samples A and B were followed by means of XPS (Figure 3). Binding energies (B.E.) of main features and atomic percentages of Mo and Ni species according to their contribution to the overall signal envelope are compiled in Table 3. For samples A (sulfide) and B (sulfide), three doublets related to different Mo oxidation states, together with the S 2s signal characteristic of S^2–^ (falling in the same spectral range at a B.E. >226.3 eV) can be identified. The three Mo 3d doublets are assigned to Mo^6+^ in MoO_3_ (B.E. 3d_5/2_ = 232.9 ± 0.4 eV), Mo^5+^ in oxysulfide species (B.E. 3d_5/2_ = 230.8 ± 0.4 eV) and Mo^4+^ in MoS_2_ (B.E. 3d_5/2_ = 229.1 ± 0.5 eV). The Ni 2p spectra can be deconvoluted into three components:^69^ oxidic Ni (856.4 ± 0.6), NiMoS species (854.6 ± 0.4 eV), and sulfided Ni (853.3 ± 0.2 eV), along with their respective satellites.^70,71^ The spectra of samples A (oxide) and B (oxide) were fitted in the same way with oxidic contributions only.

**Table 3:** Binding Energy and Atomic Percentage of Samples A (Sulfide) and B (Sulfide) Sulfided under 1 bar H_2_S/H_2_ at Various Temperatures and Their Respective Oxidic Precursors (Oxide)

		binding energy (eV)						fractions (%)					
		Mo 3d_5/2_			Ni 2p_3/2_			Mo 3d			Ni 2p		
sample	temperature (°C)	Mo^4+^	Mo^5+^	Mo^6+^	NiS*_x_*	NiMoS	NiO*_x_*	Mo^4+^	Mo^5+^	Mo^6+^	NiS*_x_*	NiMoS	NiO*_x_*
A	350	229.03	230.85	233.02	853.16	854.82	856.84	85.1	10.7	4.2	72.2	23.0	4.8
	250	228.98	230.83	232.98	853.37	854.93	856.79	73.5	16.6	9.9	68.3	19.7	12.0
	150	228.96	230.90	232.78	853.27	854.95	856.79	48.3	23.1	28.6	68.0	17.2	14.8
	50	228.95	230.88	232.27	853.21	854.99	856.73	16.7	20.6	62.7	48.7	19.1	32.2
	oxide			232.72			856.77	0	0	100	0		100
B	350	229.00	230.71	233.02	853.40	854.89	856.84	87.7	9.3	3.0	67.0	26.4	6.6
	250	229.00	230.91	233.01	853.41	854.93	856.79	73.8	16.2	10.0	65.6	25.3	9.1
	150	229.01	230.90	233.14	853.32	854.93	856.79	69.4	19.1	11.5	65.8	23.8	10.4
	50	228.98	231.07	232.72	853.32	854.96	856.84	45.4	29.9	24.7	44.0	25.8	30.2
	oxide			232.72			856.77	0	0	100	0		100

**Figure 4:**
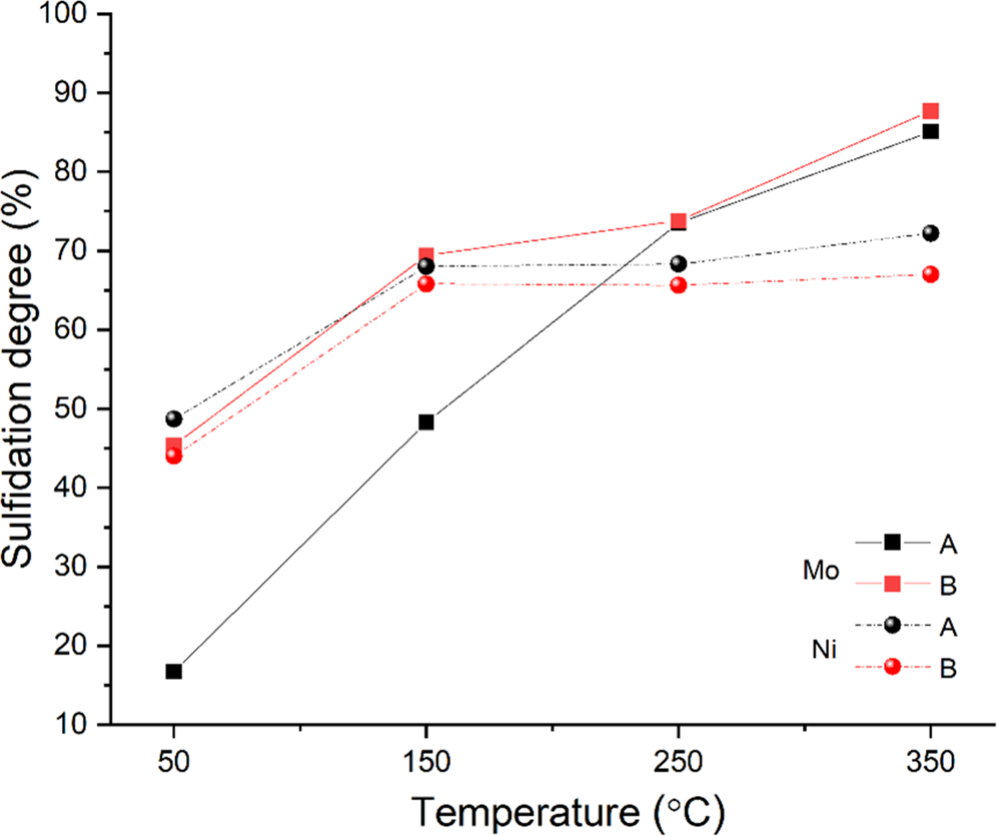
Temperature-dependent sulfidation profile of Mo and Ni in samples A and B at four different temperatures and 1 bar H_2_S/H_2_ as determined by XPS.

Figure 4 shows the sulfidation profile of samples A (sulfide) and B (sulfide). While the final sulfidation degree of Mo and Ni is comparable in both samples, the evolution toward this end value differs. For sample A (sulfide), the sulfidation rate of Ni is faster than that of Mo, from which we conclude that a substantial portion of Ni is sulfided before the formation of MoS_2_ has started. This could mean that this part of Ni remains as isolated NiS*_x_* particles and will thus not participate in the formation of the catalytically active Ni–Mo–S phase.^12,46,72^ In sample B (sulfide), the situation is reversed, i.e., Mo sulfidation precedes that of Ni, meaning MoS_2_ particles already exist when Ni sulfidation starts, providing the right environment for Ni–Mo–S formation.^73^ The concentration of NiMoS species in samples A (sulfide) and B (sulfide) as extracted from XPS (Table 3) verified this assumption.

### 3.4 Spatial Resolved Analysis of Sulfided and Spent Catalysts

To investigate how the different preparation routes affect the catalyst structure and composition locally, sulfided and spent catalysts of types A and B, were characterized by means of PXCT and STEM/EDX. This combination of techniques provides information from the micron scale (PXCT) to the nanoscale (STEM/EDX).

**Figure 5:**
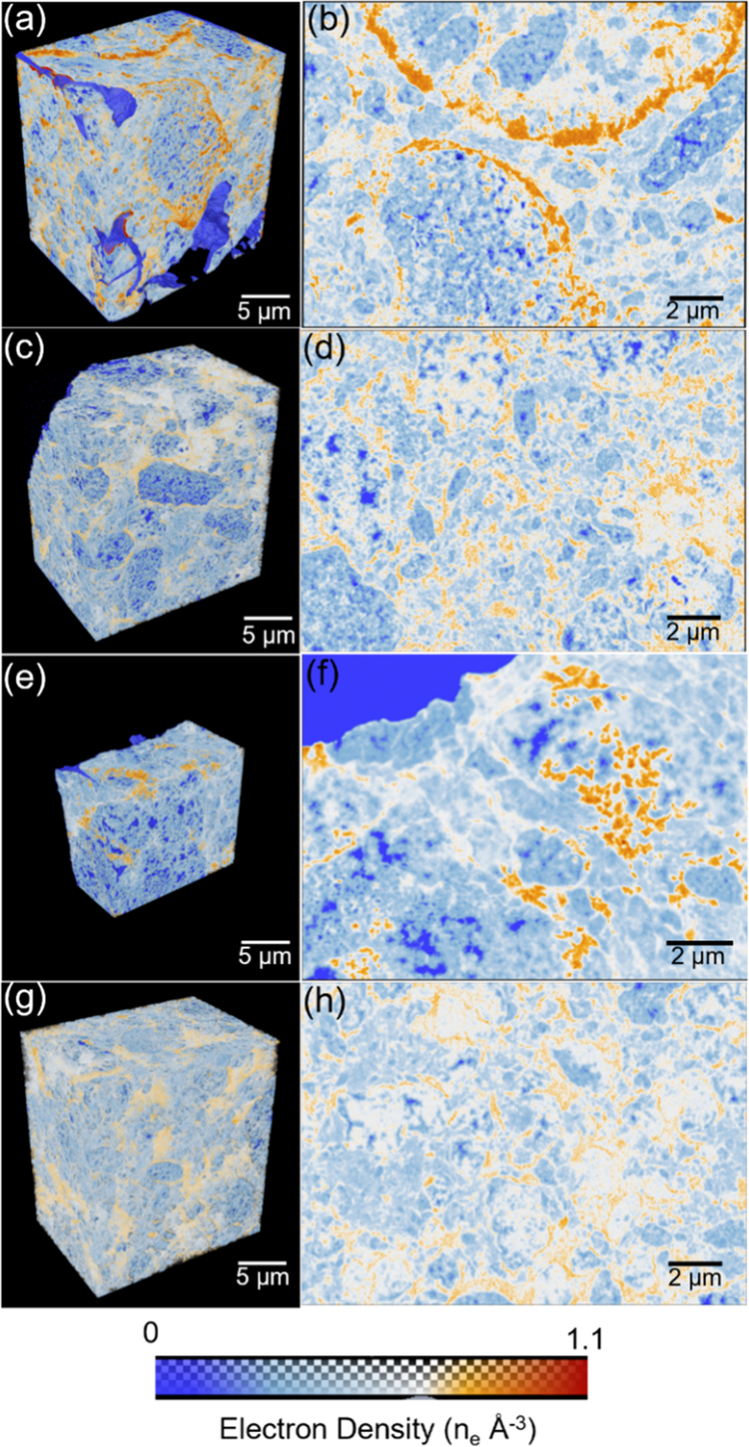
Ptychographic X-ray computed tomography of sample A (sulfide and spent) and sample B (sulfide and spent). Shown are volume renderings of and virtual cuts through the PXCT-acquired electron density tomograms of samples A (sulfide) (a, b), B (sulfide) (c, d), A (spent) (e, f), and B (spent) (g, h). The color map ranging from blue to red indicates an increase in electron density.

Figure 5 shows volume renderings and sagittal cuts through the PXCT-acquired electron density tomograms of catalyst A (sulfide and spent) and catalyst B (sulfide and spent). As the half-period spatial resolution of these tomograms is on average 40 nm (Figure S3), local or direct compositional analysis is restricted to larger mesopores (and above) and or reliant on partial volume analysis utilizing a priori compositional knowledge. Partial volume effects refer to the occupancy of a single voxel by multiple, spatially unresolved, components, leading to a fractional occupancy-related electron density.^47^ The theoretical electron densities of the main sample or catalyst components are 0.785 n_e_ Å^–3^ for amorphous Al_2_O_3_ and 1.1 n_e_ Å^–3^ for NiMoO_2_. Further information can be found in Table S1. Based on these values, we consider that an electron density below that of amorphous Al_2_O_3_ can be directly related to the degree of internal porosity. Similarly, an electron density above that of amorphous Al_2_O_3_ provides information about the amount of MoS_2_ clusters present within a selected voxel (see the Supporting Information for detailed tomogram analysis information). Adhering to this interpretation we can identify four compositionally distinct domains in the acquired electron density tomograms, which are spatially resolved pores, low-density (high internal porosity) alumina, high-density (low internal porosity) alumina, and areas rich in MoS_2_ species, >30 vol %.

A metal deposition difference can be observed between samples A (sulfide) and B (sulfide) (Figure 5a–d). Visible in sample A (sulfide), are isolated areas or clusters rich in MoS_2_ species (dark orange dots) and continuous circular domains rich in MoS_2_ species, potentially caused by a diffusion-limited drying process and reflective of metal aggregation. These domains are absent in sample B (sulfide), displaying a more homogeneous distribution of MoS_2_. Further evidence of this can be found in the fact that the smaller clusters present in sample B have a lower electron density compared to sample A, i.e., the corresponding voxel possesses a lower MoS_2_ concentration.

**Figure 6:**
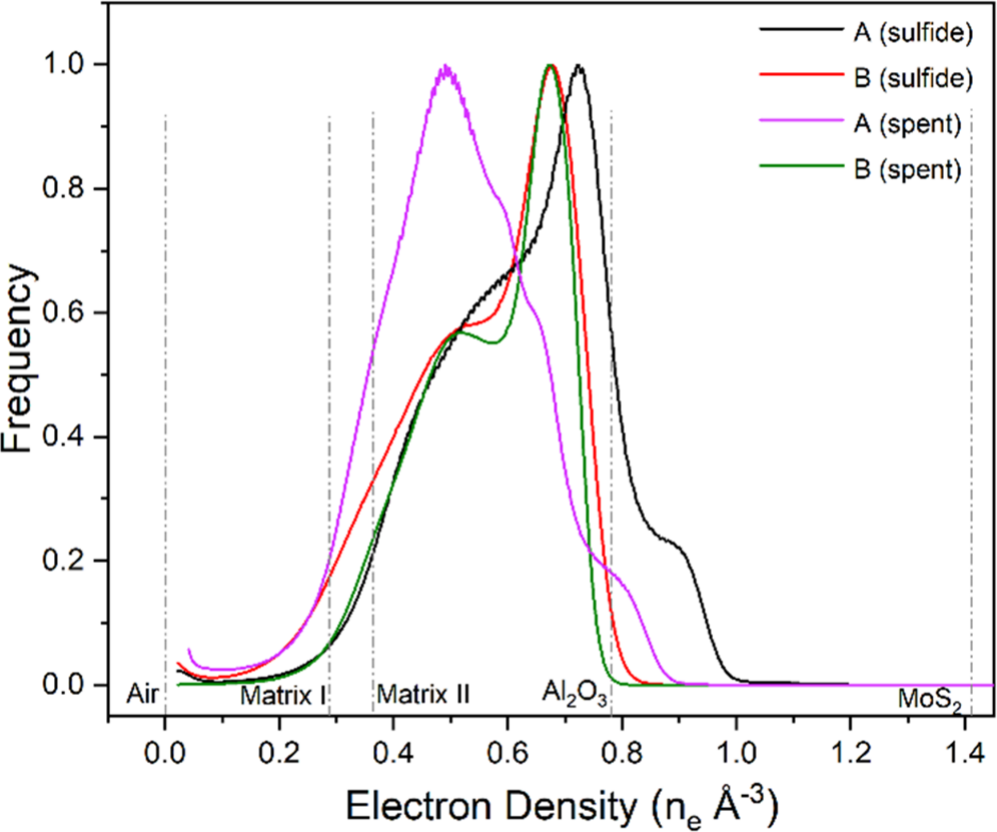
Tomogram corresponding electron density histograms of sample A (sulfide and spent) and sample B (sulfide and spent). The electron density of selected reference components is indicated by the dotted lines.

This metal deposition behavior is also found in the tomogram corresponding electron density histograms (Figure 6). Here, we see a shoulder in the high-electron-density region (bottom right area) for sample A (sulfide); while in sample B (sulfide), no such high-electron-density area is observed. From the PXCT data, we can infer that the metal dispersion in sample B (sulfide) is much higher than in sample A (sulfide). Looking at the sagittal cuts through the tomograms of these two samples, it can also be noted that the above-mentioned high-electron-density and MoS_2_-rich regions did not uniformly form throughout the alumina domain. Visible in the sagittal cuts is a diminishing electron density gradient from the larger pore space into high-density alumina domains. This observed electron density gradient can result from particle aggregation at the boundary of high- and low-density alumina due to metal deposition limitation encountered upon entering these domains, i.e., a result of pore transport limitations.^47^ These limitations appear to be much stronger in sample A (sulfide). No such gradient is observable in the low-density or high-porosity alumina domains.

Focusing on the spatially resolved pore structure we observe further differences between these two samples (Figure S5). While the pores in sample B (sulfide) have a diameter between 60 and 420 nm, the pores found within sample A (sulfide) are slightly larger with a diameter distributed in the range of 60–760 nm. As the compositions of catalyst and support material are the same, we infer that this difference was caused by specific interactions of the impregnation solution with the alumina carrier, resulting in alterations of the pore structure. The unchanged pore size distribution between the sulfide and their respective spent samples (Figure S5) provides further evidence that structural differences of the carrier between samples A (sulfide) and B (sulfide) were introduced during catalyst preparation. Moreover, it demonstrates that liquid phase sulfidation used in the gas oil test does not change the hierarchical structure of the catalysts, which makes a comparison between sulfide samples (gas phase sulfidation) and spent samples (liquid phase sulfidation) used for stability study, reasonable.

As shown in Figures 5 and 6, the electron densities of samples B (sulfide) and B (spent) are comparable, indicating that activity test conditions did not cause much difference to the structure of sample B (sulfide). For sample A we do observe a significant change following the activity test. Visible is a decrease of the high-electron-density components, the histogram peak previously associated with MoS_2_ cluster changes in position from 0.9 to ∼0.8 n_e_/Å^3^ and decreases in relative intensity. To understand why the high-electron-density region decreases, elemental analysis was conducted (Table 1). The results show that the metal contents in sample A (spent) (9.16 wt % Mo, 2.57 wt % Ni) is lower than in A (sulfide) (10.42 wt % Mo, 2.81 wt % Ni), from which we conclude that metal, presumably in the form of MoS_2_ species, was washed off under the gas oil activity test conditions.

As the here acquired PXCT data are limited in spatial resolution and chemical element insensitive, we mechanically fractured the catalyst pellets to obtain an electron-microscopy-compatible specimen. Subsequent examination using STEM and EDX mapping allowed us to probe the effect preparation, activation and use had on the structure of the active MoS_2_ platelets as well as the catalyst’s local elemental composition.

**Figure 7:**
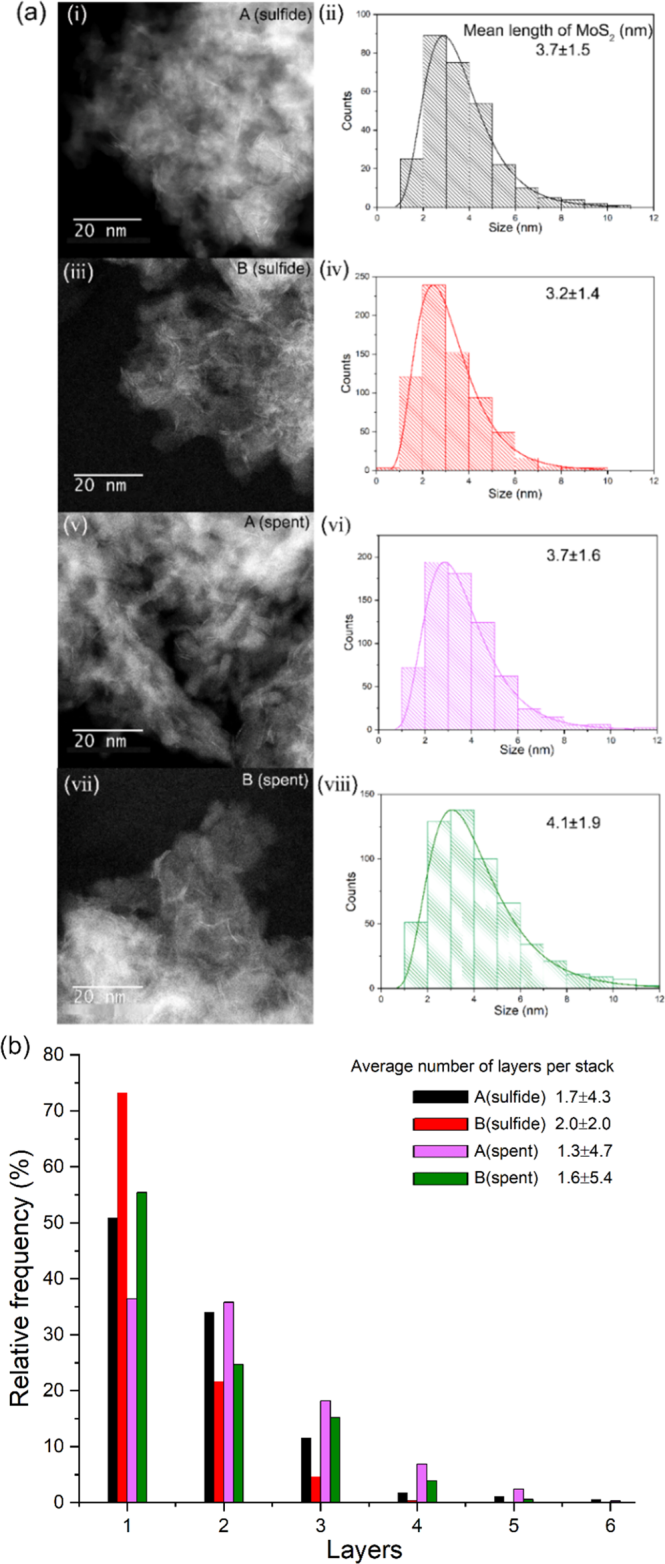
(a) High-angle annular dark-field (HAADF) STEM images and length distribution of MoS_2_ platelets of (i, ii) A (sulfide), (iii, iv) B (sulfide), (v, vi) A (spent), and (vii, viii) B (spent); (b) stacking distribution and the average number of layers per stack of four prepared samples (A (sulfide), B (sulfide), A (spent), and B (spent)).

Figure 7a shows STEM images of samples A and B and provides information on the length distribution of MoS_2_ platelets as well as their layers distribution. The mean length of MoS_2_ platelets in sample A (sulfide) is 3.7 nm with an average 1.7 layers per particle; particles in sample B (sulfide) are smaller (3.2 nm) with a higher number of layers (2.0). The feature of small particle size and high degree of stacking per particle is often considered a prerequisite to the formation of type II NiMoS phases.^46^

While the activity test had no effect on the mean length of MoS_2_ platelets in sample A (spent), a slight increase in mean length can be observed in sample B (spent). Additionally, a notable de-stacking was observed: the average number of layers per particle decreased from 1.7 (A (sulfide)) to 1.3 (A (spent)) and from 2.0 (B (sulfide)) to 1.6 (B (spent)), Figure 7b. De la Rosa et al.^74^ assign this de-stacking behavior during catalyst operation to the pressure applied in the HDS activity test, since at high pressure, the formation of multilayered stacks through van der Waals forces seems counterbalanced by the strong interaction of adsorbed substances favoring stabilization of single MoS_2_ layers.

**Figure 8:**
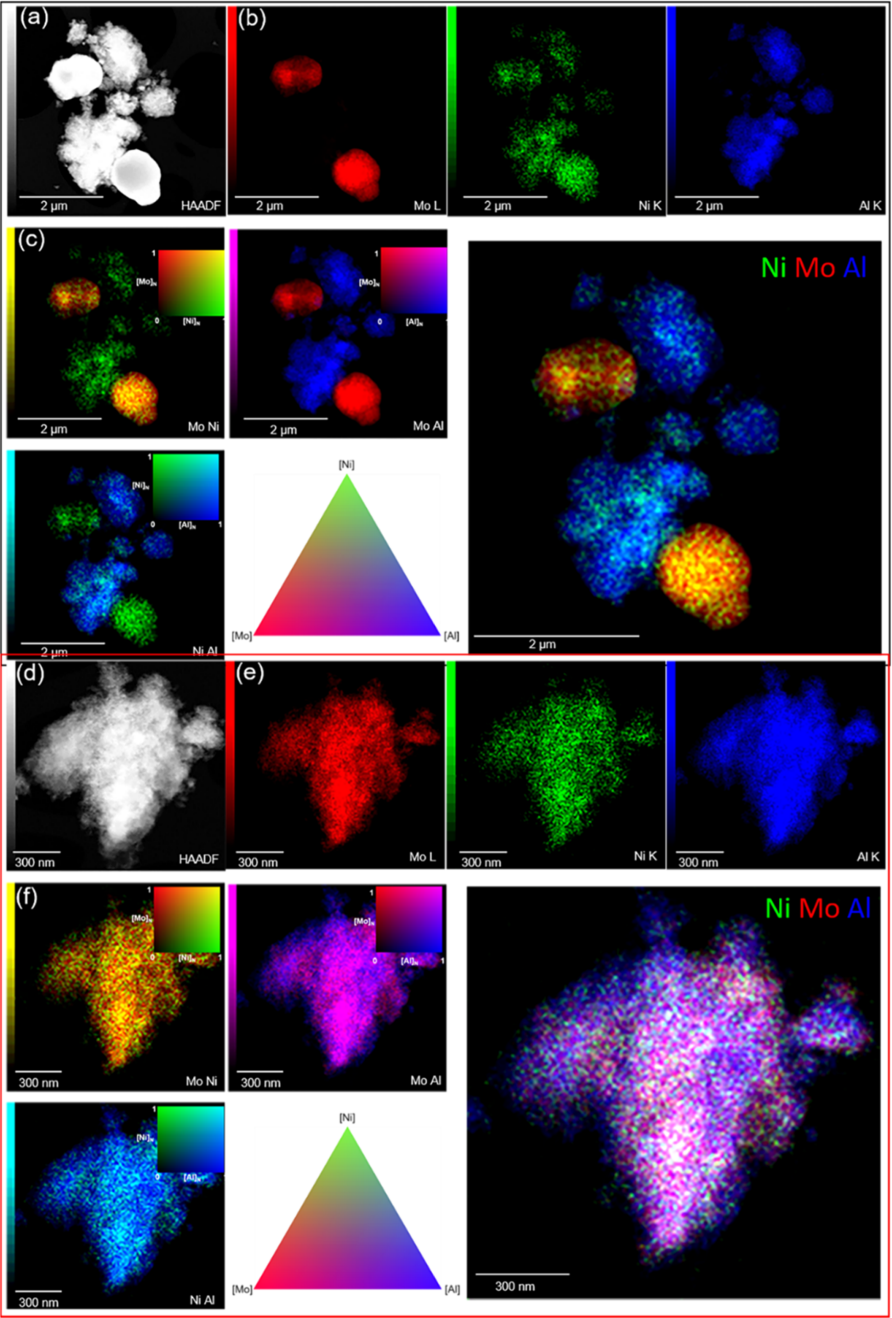
Electron microscopy of A (sulfide) (top) and B (sulfide) (bottom). (a, d) High-angle annular dark-field (HAADF) STEM image. (b, e) Energy-dispersive X-ray spectroscopy (EDX) maps of Mo, Ni, and Al corresponding to the STEM image of (a) and (d). (c, f) Normalized (N) and merged EDX maps of Mo, Ni, and Al.

To probe the elemental distribution in the prepared catalysts on the nanoscale we referred to a combination of HAADF STEM and EXD mapping. Figure 8a shows the HAADF STEM image revealing the presence of two morphologically distinctly different phases in sample A (sulfide). EDX-derived compositional maps of Mo, Ni, and Al (Figure 8b,c) suggest these phases to correspond to unsupported NiMoS particles and isolated NiS*_x_* species. As suggested by XPS, this could be the result of the faster sulfidation rate of Ni under the chosen preparation conditions. In sample B (sulfide), the metals are dominantly homogeneously dispersed as expected for Ni–Mo–S particles. The spatial correlation of Ni and Mo, i.e., their dispersion behavior, can be quantified using a normalized Ni–Mo correlation degree extracted from EDX mapping data, as shown in Figure S8. Here it is shown that the average correlation degree in sample A (sulfide) is 0.7, while it is 0.9 in sample B (sulfide). After the gas-oil test, the average Ni–Mo correlation degree, for samples A (spent) and B (spent), decreased to 0.7 and 0.5, respectively. The origin of this decrease could be owed to an aggregation of MoS_2_ and NiS*_x_*, during the gas oil activity test (see EDX data of samples A (spent) and B (spent) in Figures S6 and S7). Under the testing conditions, NiS*_x_* will have sufficiently high mobility to enable aggregation.^75^

## 4 Discussion

The comparative analysis of two “*compositionally*” identical alumina-supported NiMo hydrotreating catalysts, originating from different preparation methodologies, revealed pronounced differences in both catalytic activity and stability. Examination of the supported catalysts in the oxidic precursor state, the active sulfide, and post-use or spent state revealed these differences to be a result of compositional and structural modifications of both the support and the catalyst.

The oxidic precursors were characterized by means of TPR, XRD, and Raman spectroscopy. The results indicate that in samples A and B, Mo and Ni species exist on the alumina carrier surface in different coordination states. As a consequence, samples A and B exhibit varying degrees of metal–support interactions (Figures 1 and 2) and ultimately a distinct response to hydrogen reduction, as well as sulfidation, i.e., to the transformation of Ni–Mo–O into Ni–Mo–S phases. XPS results (Figures 3 and 4) show that both samples arrive at the same degree of Mo and Ni sulfidation at the end of the sulfidation process; however, the evolution toward the state is different. In sample A (sulfide), most part of Ni is sulfided before the pronounced formation of MoS_2_ has started, so it is likely this part of Ni remains as isolated NiS*_x_* particles and will not participate in forming the catalytically active Ni–Mo–S phase. This interpretation is also supported by STEM/EDX data, which show unsupported MoS_2_ particles and isolated NiS*_x_* species (Figure 8b,c). Sample B (sulfide) behaves differently; MoS_2_ particles already exist when Ni sulfidation starts, and incorporation of Ni into the edges of existing MoS_2_ particles is the predominant utilization of Ni, yielding an enhanced formation of NiMoS particles (higher content), which is in line with the distribution pattern of Ni and Mo shown in STEM/EDX (Figure 8e,f). This structure can be further verified via the normalized Ni–Mo correlation degree (Figure S8), extracted from the EDX mapping data, since a higher average degree of Ni–Mo correlation detected in sample B (sulfide) is the expected appearance for a sample consisting predominantly of NiMoS particles. This difference in sulfidation chemistry indicates that sample A (oxide) has a higher metal–support interaction than sample B (oxide), and this is reflected as a lower degree of stacking and larger MoS_2_ particles in sample A (sulfide) as observed by STEM (Figure 7).

Different metal coordination and metal–support interactions in the oxidic precursors are not the only changes caused by preparation conditions; changes in the (pore)structure of the alumina carrier are also observed. Pore diameter distribution values extracted from PXCT data (Figure S4) show that sample A (sulfide) has larger pores compared to sample B (sulfide), most likely formed as the result of partial alumina dissolution during the preparation (impregnation) process. The pore diameter data derived from BET measurements underpin this assumption (Table 1); sample A (oxide) has a larger pore diameter than sample B (oxide). The alumina dissolution process can be considered to be a consequence of the formation of heteropolymolybdate, as initially proposed by Carrier et al.^76^ They conclude that alumina dissolution occurs in the presence of molybdates, and especially in the pH range between 4 and 6. As mentioned in Section 2.2, the pH value of impregnation solution A is around 5 and does thus fall into this pH range, whereas the pH of solution B is <1. However, the pore size distribution of the sulfide samples with their respective spent samples is comparable.

**Scheme 1:**
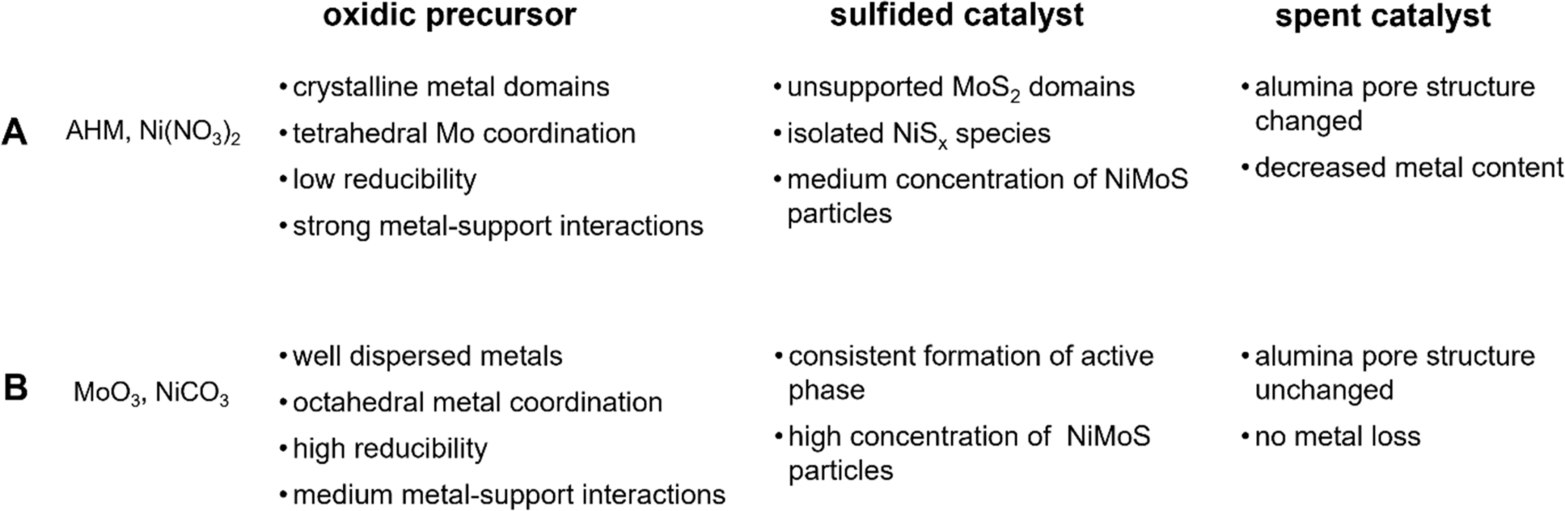
Summary of Samples Properties

Industrial hydrotreating catalysts are prepared and delivered as oxidic precursors, which makes it necessary to understand their structure. However, true understanding of catalytic performance lies in characterizing the structure of the active sulfides and relating their structures to performance data. To understand the 3D structure of our two sulfided samples, we used PXCT measurements to reveal metal dispersion and pore structure at the micrometer scale. PXCT is a technology that provides information on electron density differences in the measured samples. By comparing volume rendering and sagittal cuts of samples A (sulfide) and B (sulfide) (Figure 5), major differences can be seen; the uniform distributed electron density indicates that metals are well dispersed in sample B (sulfide), while for sample A (sulfide), besides the part of the well-distributed metals, two remarkable circular areas with high electron density, likely regions of increased metal (MoS_2_) concentration can also be observed. However, based on the PXCT data, it cannot be decided if metal aggregation processes cause this high electron density. To get a better understanding of that, we performed STEM/EDX, where large unsupported MoS_2_ and isolated NiS*_x_* patches were detected in sample A (sulfide) (Figure 8a). Hence, it is reasonable to consider that this part of metals, which according to our XPS data did not participate in the formation of Ni–Mo–S particles, exists as isolated MoS_2_ and NiS*_x_* species. Likely, these species aggregate under operation conditions; similar types of larger, isolated MoS_2_ and NiS*_x_* patches are observed in samples A (spent) and B (spent) (Figures S6 and S7). Furthermore, an obvious difference in electron density between samples A (sulfide) and A (spent) observed in the extracted PXCT data (Figures 5a,b,e,f and 6) indicates that metal domains in sample A (sulfide) are not stable under operation conditions. To verify if this change is due to a change of metal concentration during the catalytic activity test, we determined metal concentrations in the fresh sulfided and spent samples by means of ICP measurements (Table 1). Sample A (spent) contains 12.1% less Mo and 9% less Ni than sample A (sulfide), which means that this portion was washed off during the activity test. This was different for sample B; ICP data show similar metal contents in B (sulfide) (10.70 wt % Mo, 2.56 wt % Ni) and B (spent) (10.69 wt % Mo, 2.57 wt % Ni). Here, PXCT and STEM/EDX results both consistently show a Ni and Mo distribution pattern in line with the existence of NiMoS particles and confirm our interpretation of the XPS data that the metals are largely leveraged into forming active and sufficiently stable NiMoS particles. A summary of sample properties and differences is given in Scheme 1.

## 5 Conclusions

We have investigated two HDS catalysts (A and B) that are compositionally identical, same alumina carrier, organic additive, metal, and phosphorus loading, but that are prepared under different conditions, i.e., different sources of Mo and Ni were used for the impregnation solutions. While the impregnation solution of sample A was made from (NH_4_)_6_Mo_7_O_24_ and Ni(NO_3_)_2_ as metal sources, MoO_3_ and NiCO_3_ were used in the case of sample B. The structural differences between these two catalyst samples, caused by changes in preparation conditions, were studied in detail by characterizing the oxidic precursors, the sulfided and the spent state of these catalysts by means of TPR, XRD, Raman spectroscopy, PXCT, STEM, and EDX. Our results indicate that the alumina pore structure is quite different between these two samples after impregnation and larger pores are formed in sample A (oxide) during preparation. Further, sample B (oxide) has a higher reducibility of the metal species and a weaker metal–support interaction. After sulfidation, the metals are well dispersed on the alumina carrier surface and primarily occur as active NiMoS particles, which are stable and do not visibly change during operation. Sample A (sulfide), however, is very different. Only part of the metal converts into active NiMoS particles, while the remaining metal occurs as unsupported MoS_2_ and isolated NiS*_x_* clusters, which are either washed off during operation or aggregate into larger, unsupported NiMo domains. This structural description explains the enormous activity differences between these two samples and explains why sample B is much more active than sample.

In this work, we revisited a problem of catalyst preparation and identified a critical performance driver that was largely overlooked in many studies, i.e., the chemicals used to design an impregnation solution. Not only could we show that impregnation solutions based on (NH_4_)_6_Mo_7_O_24_—a source of molybdenum used in many studies—yield catalysts of low performance, but we were also able to explain this finding by structural properties and visualize those in the 3D space. Pushing catalyst innovation in a mature technology like hydrodesulfurization is challenging and requires in-depth understanding of catalyst activity relations across different length scales of catalyst structure.

## Supporting Information Available

Volume rendering ptychographic tomograms; shown are volume reconstructions of and cut slices through the retrieved electron density tomograms of sample A (sulfide) (AVI)
Volume rendering ptychographic tomograms; shown are volume reconstructions of and cut slices through the retrieved electron density tomograms of sample B (sulfide) (AVI)
Volume rendering ptychographic tomograms; shown are volume reconstructions of and cut slices through the retrieved electron density tomograms of sample A (spent) (AVI)
Volume rendering ptychographic tomograms; shown are volume reconstructions of and cut slices through the retrieved electron density tomograms of sample B (spent) (AVI)
Extended methods section regarding PXCT sample preparation, data acquisition, data reconstruction, the methods for resolution and dose evaluation as well as tomograms and EDX analysis; physisorption results of two oxides; SEM of one of the examined NiMo catalyst pillars; Fourier shell correlation (FSC) line plots of the electron density tomograms of examined catalyst pillars; Raman spectra of two respective impregnation solutions; normalized frequency pore size distributions for samples A (sulfide), B (sulfide), A (spent), and B (spent); high-angle annular dark-field (HAADF) STEM image and energy-dispersive X-ray spectroscopy (EDX) maps of molybdenum (Mo), nickel (Ni) and aluminum (Al) corresponding to the STEM image of samples A (spent) and B (spent); and normalized Ni–Mo correlation degree of samples A (sulfide), B (sulfide), A (spent) and B (spent) extracted from EDX mapping data (PDF)

## Supplementary Material

jp2c05927_si_001.avi

jp2c05927_si_002.avi

jp2c05927_si_003.avi

jp2c05927_si_004.avi
