# Supplementary material for: Alumina-Supported NiMo Hydrotreating Catalysts—Aspects of 3D Structure, Synthesis, and Activity
Source: J Phys Chem C Nanomater Interfaces. 2022 Oct 24;126(43):18536–49. doi: 10.1021/acs.jpcc.2c05927 (PMC9639170; doi:10.1021/acs.jpcc.2c05927)
Supplement: Supplementary file 5 — jp2c05927_si_005.pdf [file jp2c05927_si_005.pdf]

# Supporting Information

Alumina Supported NiMo Hydrotreating Catalysts - Aspects of 3D Structure, Synthesis and Activity

*Mengyan Li* [a], *Johannes Ihli* [b], *Marcel A. Verheijen* [c], *Mirko Holler* [b], *Manuel Guizar-Sicairos* [b], *Jeroen Anton van Bokhoven* [b, d], *Emiel J.M. Hensen* [a], *Thomas Weber* [a,*]

a. Laboratory of Inorganic Materials and Catalysis, Department of Chemical Engineering and Chemistry, Eindhoven University of Technology, Het Kranenveld 14, 5600 MB, Eindhoven, The Netherlands

b. Paul Scherrer Institute, 5232 Villigen PSI, Switzerland

c. Department of Applied Physics, Eindhoven University of Technology, Eindhoven 5600 MB, The Netherlands; Eurofins Materials Science, Eindhoven 5656 AE, The Netherlands

d. Institute for Chemical and Bioengineering, Department of Chemistry and Applied Biosciences, ETH Zurich, Vladimir-Prelog-Weg 1–5/10, 8093 Zurich (Switzerland)

Email: Th.Weber@tue.nl

## METHOD

**PXCT sample preparation and mounting.** Catalyst pellets were first mechanically fractured, then a central piece from each pellet was mounted on a tomography pin[1]. The mounted samples were then pre-shaped using a micro-lathe[2] to a diameter of 80-50 µm before being reduced to < 25 µm in diameter using focused ion-beam (FIB) milling. See Figure S1 for a scanning electron micrograph of one of the prepared pillars. The prepared samples were stored in evacuated desiccators prior to the measurement to avoid moisture contamination.

**Data acquisition and reconstruction.** PXCT experiments were carried out at the cSAXS beamline of the Swiss light source (SLS). The photon energy was selected using a double-crystal Si(111) monochromator. Measurements were carried out at 6.2 keV. A Fresnel zone plate with 200 µm diameter, outermost zone width of 60 nm, and locally displaced zones to provide perturbations of the illumination wavefront, was used to define the illumination onto the sample[3]. The sample was placed ~1.5 mm downstream of the zone plate's focal point. Coherent diffraction patterns were acquired with an in-vacuum 1.5k Eiger detector with a 75 µm pixel size approximately 7.2 m downstream of the sample[4-5]. The sample to detector distance was 5.23 m. An evacuated flight tube was positioned between the sample and detector to reduce air scattering and absorption. Measurements were carried out using the positioning instrumentation described in Holler et al[6-8]. at room temperature under a nitrogen dome.

Scanning positions for each tomographic projection were set using a Fermat spiral [9] scanning grid with an average step size of 1 micron. The total field of view covered in a projection was at maximum 30 by 15 µm. Depending on the sample diameter and apparent achievable resolution, based of Fourier ring correlation of tomographic projections, between 650-1423 projections were acquired and later used for the tomographic reconstruction. The acquisition time per scanning point

in a single projection was 0.025 seconds. From each of these diffraction patterns, a region of 600 × 600 detector pixels was used for the ptychographic reconstructions. Projection reconstructions were obtained with 300 iterations of the difference map algorithm followed by 500 iterations of maximum likelihood refinement, using the PtychoShelves package[10]. Projections were acquired, aligned and preprocessed using the approach in [11]. The projections were aligned, and complex-valued 3D index of refraction were computed using a modified filtered back-projection algorithm (FBP). The alignment step of projections is important for achieving high resolution in 3D. The resulting tomograms possess a voxel size of $(23.26\ \mathrm{nm})^3$.

**Resolution evaluation.** The half-period spatial resolution of ptychographic tomograms was estimated using Fourier shell correlation (FSC) with the ½-bit threshold criteria[12]. FSC line plots are shown in Figure S2.

**Dose evaluation.** The X-ray dose imparted to the imaged specimens during tomogram acquisition was estimated to be on the order of ~ $5e^{10}$ Gy. This was computed using the surface-dose formula in [13], using the area flux density during each ptychographic scan and the mass density of the specimen into consideration.[13] The specimen was assumed to consist of alumina.

**Tomogram analysis.** Analysis, segmentation, and 3D rendering was carried out using Avizo and Fiji[14]. Due to the good spatial-resolution and signal-to-noise ratio, the analysis was limited to the phase or the electron density tomograms[15]. The real part of the scattering factor  associated with the retrieved phase of $Al_2O_3$, i.e. the main sample component, is ~50x larger compared to the imaginary part of the scattering factor associated with the absorption. Further, to exclude any potential sample preparation artefacts near the edges we virtually extracted, roughly equal sized, sub-volumes from the centre of each of the imaged sample volumes.

Component identification and subsequent segmentation was achieved by comparing calculated electron densities of known catalyst components, e.g., air, $Al_2O_3$, $MoS_2$, with measured electron densities. Provided in Table S1 are electron densities of known catalysts and reference compounds. Tomograms were component segmented by interactive thresholding and followed by morphological operations to refine the segmentation further. To isolate the present macropores we selected an electron density threshold of 0.156 $n_e$ $Å^{-3}$. Assuming a binary composition of air and amorphous $Al_2O_3$ the selected cut-off value considers voxels with a composition of > 80% pores and < 20 $Al_2O_3$ to be pores. To isolate the two $Al_2O_3$ phases (high and low micro- and meso-porosity) we applied a threshold of 0.32 $n_e$ $Å^{-3}$. The contribution of monolayer $MoS_2$ per voxel to electron density was also considered, it shows that the electron density will increase by 3% with the addition of one monolayer $MoS_2$, which is negligible compared to the contribution of $Al_2O_3$ domain, given over 90% electron density was contributed by matrix. Hence, with the overall consideration and to simplify the segmentation process for isolating two $Al_2O_3$ phases, the component of $MoS_2$ was not included. Lastly, to visualize cluster of $MoS_2$, we applied an electron density threshold of 0.96 $n_e$ $Å^{-3}$. Assuming a binary composition of amorphous $Al_2O_3$ the selected cut-off value highlights cluster that are at minimum composed to 30 vol.% $MoS_2$. The isolated pore-networks were further subjected to 3D thickness map calculations to extract pore size distributions (PSD), see Figure S3. Pores with a diameter smaller than 60 nm were excluded from the shown size distributions, in view of resolution limits. Although the spatial resolution of the tomograms is insufficient to resolve individual $MoS_2$ monolayers, using the known densities of both $MoS_2$ and $Al_2O_3$, we can convert voxel-level electron density values to an approximate volumetric or mass fraction $MoS_2$ cluster concentration[16]. To note, we neglect the added Ni in this approximation, in view of the minor presence in the catalyst, and the possible contribution of pores

in this partial volume based analysis. As such the reported $MoS_2$ cluster concentrations are representative of their maximum concentration.

**Energy dispersive X-ray (EDX) image analysis**

Around 5 EDX images were used for image analysis for each catalyst. In order to use numerical values to express the correlation between Mo and Ni more directly, instead of just using the visual impressions, a programmed algorithm was coded in MATLAB. The number of pixels in all images is 218 × 226. For the calculation, an area of interest is chosen showing the catalyst. Then the numerical value for two elements on each pixel are compared to each other and a statistical correlation between the values of two elements for the entire image is calculated. This correlation ranges from -1 to 1, meaning totally uncorrelated to fully correlated respectively. This calculation is carried out for each element combination, including the correlation between Mo and Ni (Figure S6).

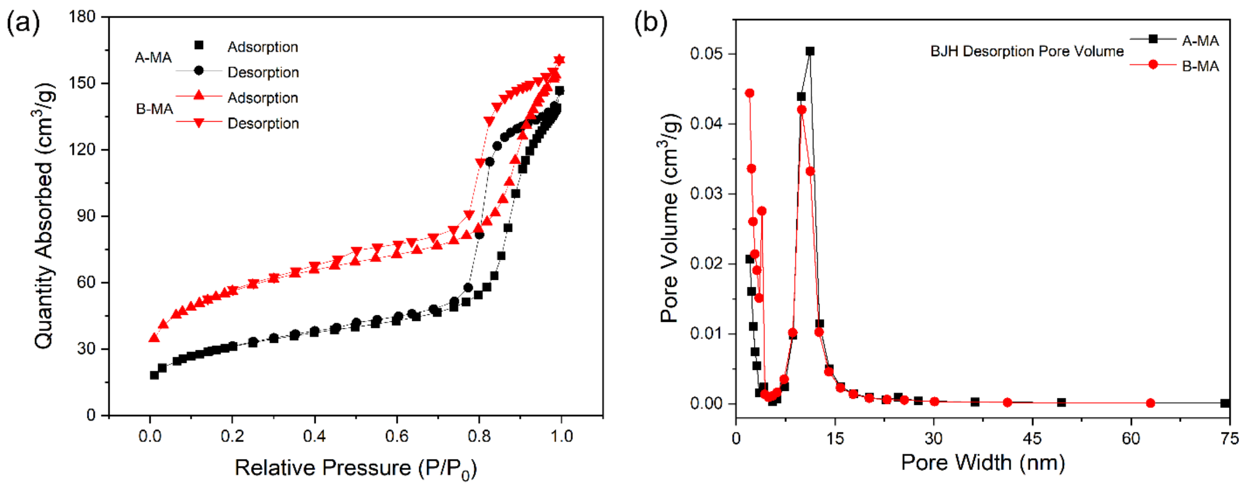


**Figure S1.** (a) $N_2$ adsorption-desorption isotherm of samples A(oxide) and B(oxide); (b) Pore size analysis results from isotherms shown in (a).

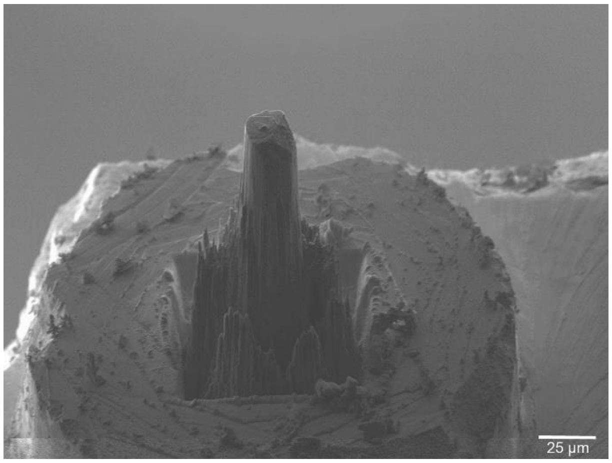


**Figure S2.** Scanning electron micrograph (SEM) of one of the examined NiMo catalyst pillars.

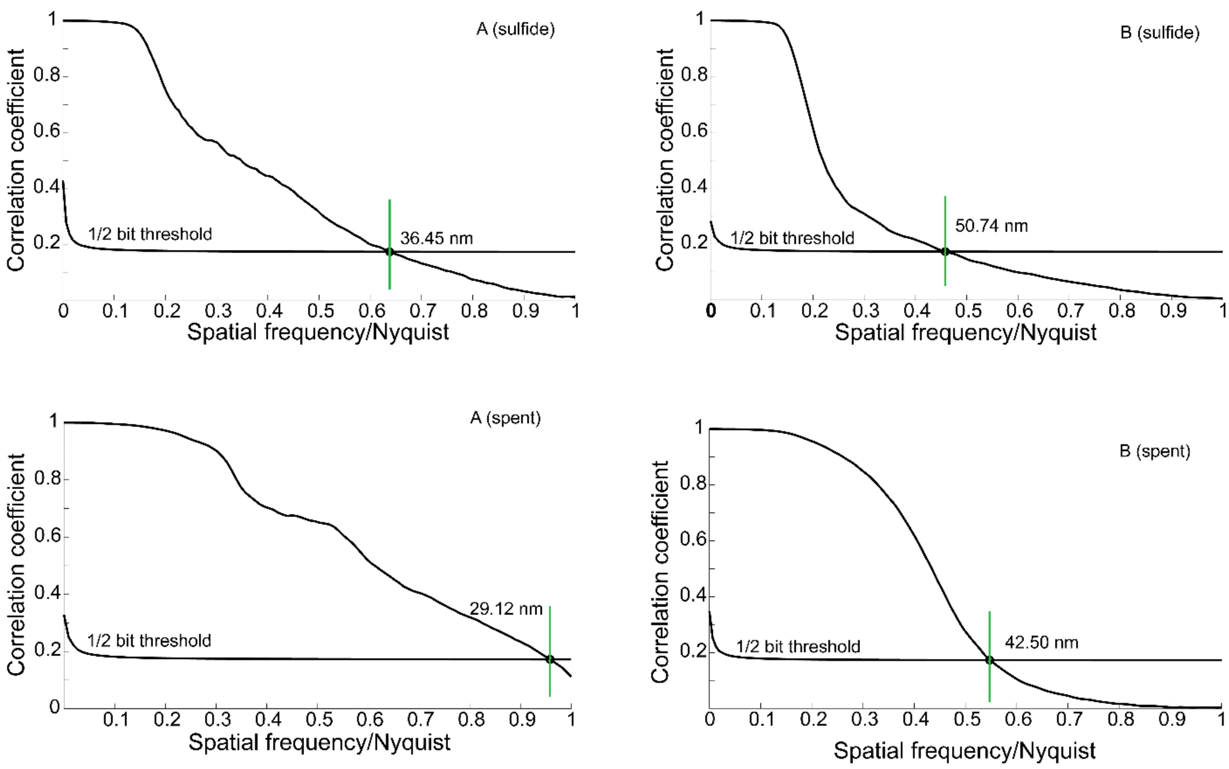


**Figure S3.** Fourier shell correlation (FSC) line plots of the electron density tomograms of examined catalyst pillars acquired at 6.2 keV. The selected threshold for determining the half-period spatial resolution was the ½ bit criterion. The Voxel size for all tomograms is $(23.26\ nm)^3$. The intersection of correlation profile and the selected threshold (green line) provides an estimate of the achieved spatial resolution. The full-period resolution is twice the half-period resolution estimates.

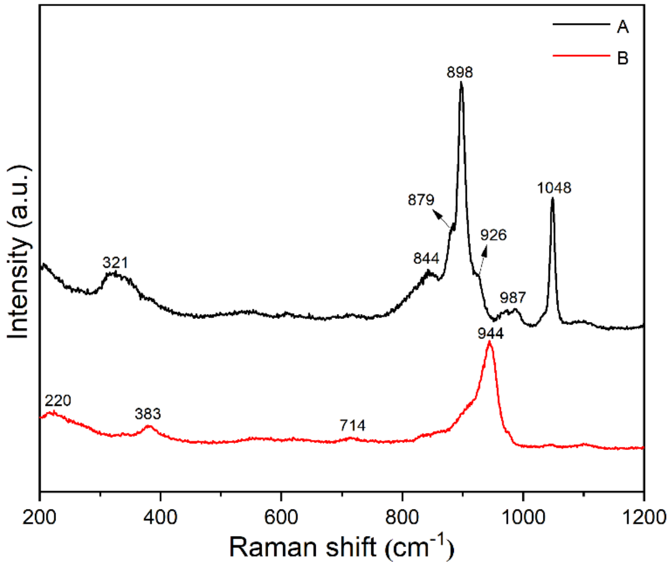


**Figure S4.** Raman spectra of two respective impregnation solutions.

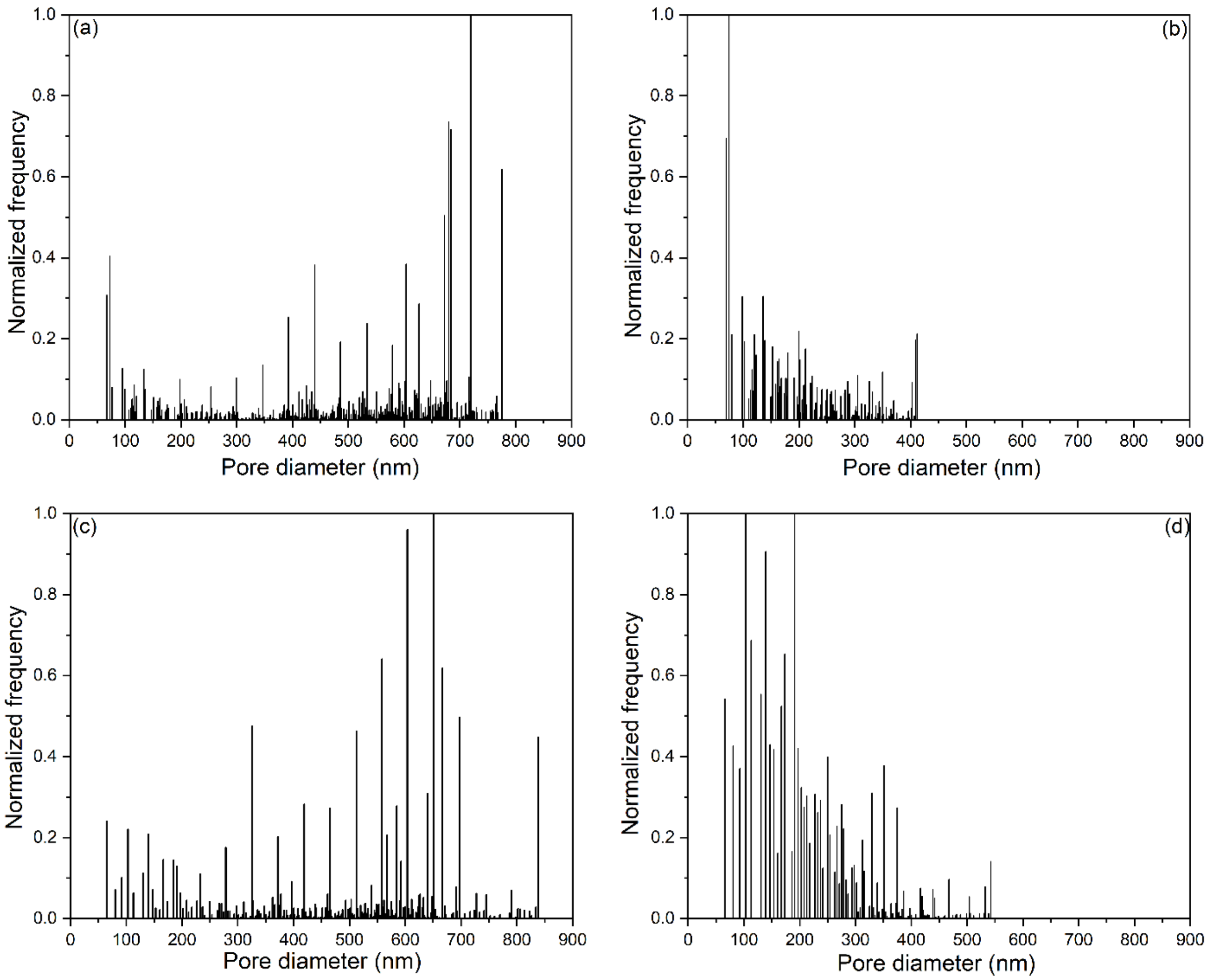


**Figure S5.** Pore size distribution of PXCT extracted, spatially resolved, pores. Shown are normalized frequency pore size distributions for samples (a) A (sulfide), (b) B (sulfide), (c) A (spent), and (d) B (spent). Only pores with a diameter greater 60 nm were considered for analysis. The respective relative volume of spatially resolved pores in samples A (sulfide), B (sulfide), A (spent) and (d) B (spent) is 1.44%, 2.3%, 0.63% and 0.14%.

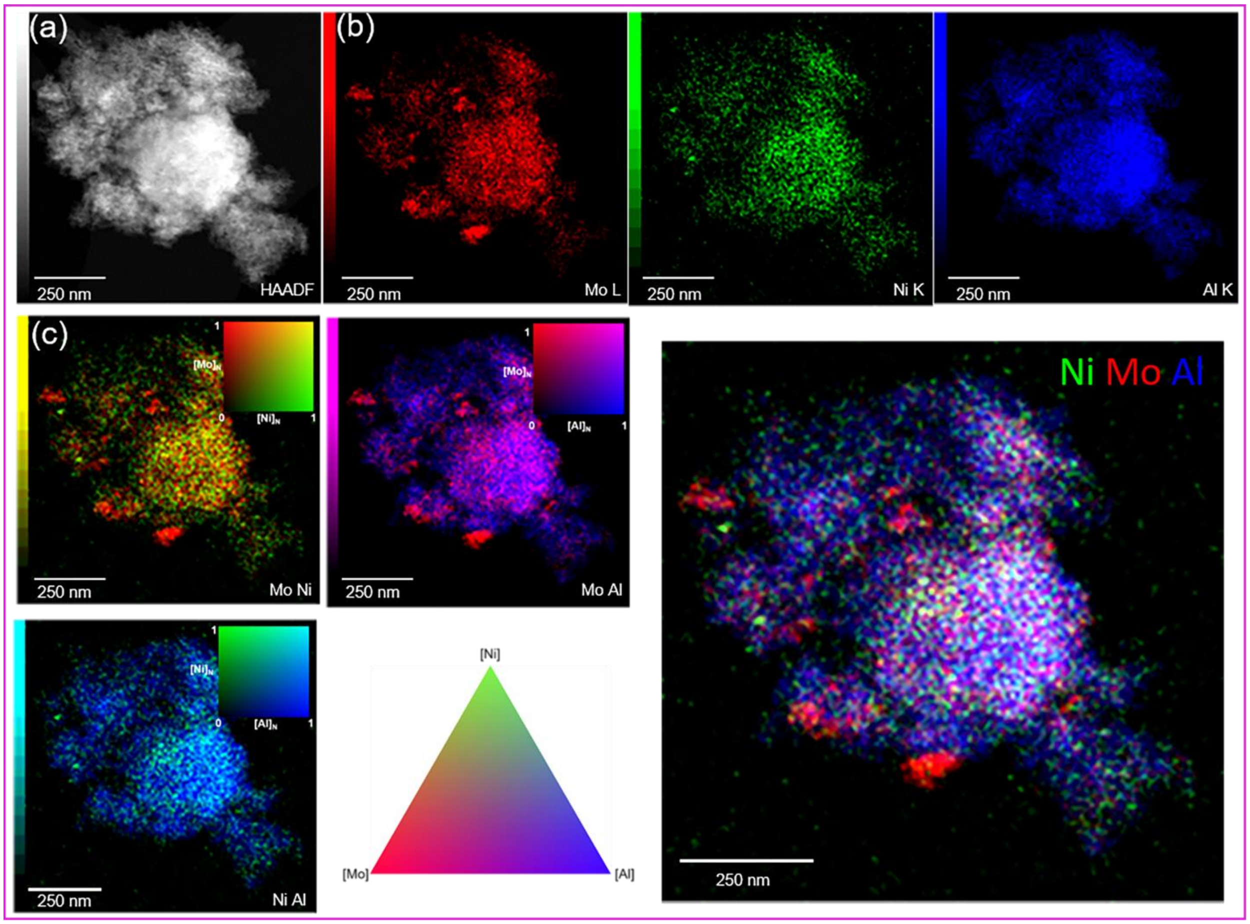


**Figure S6.** Electron microscopy of sample A (spent). a) High-angle annular dark-field (HAADF) STEM image. b) Energy dispersive X-ray spectroscopy (EDX) maps of molybdenum (Mo), nickel (Ni) and aluminum (Al) corresponding to the STEM image of (a). c) Normalized (N) and merged EDX maps of Mo, Ni and Al.

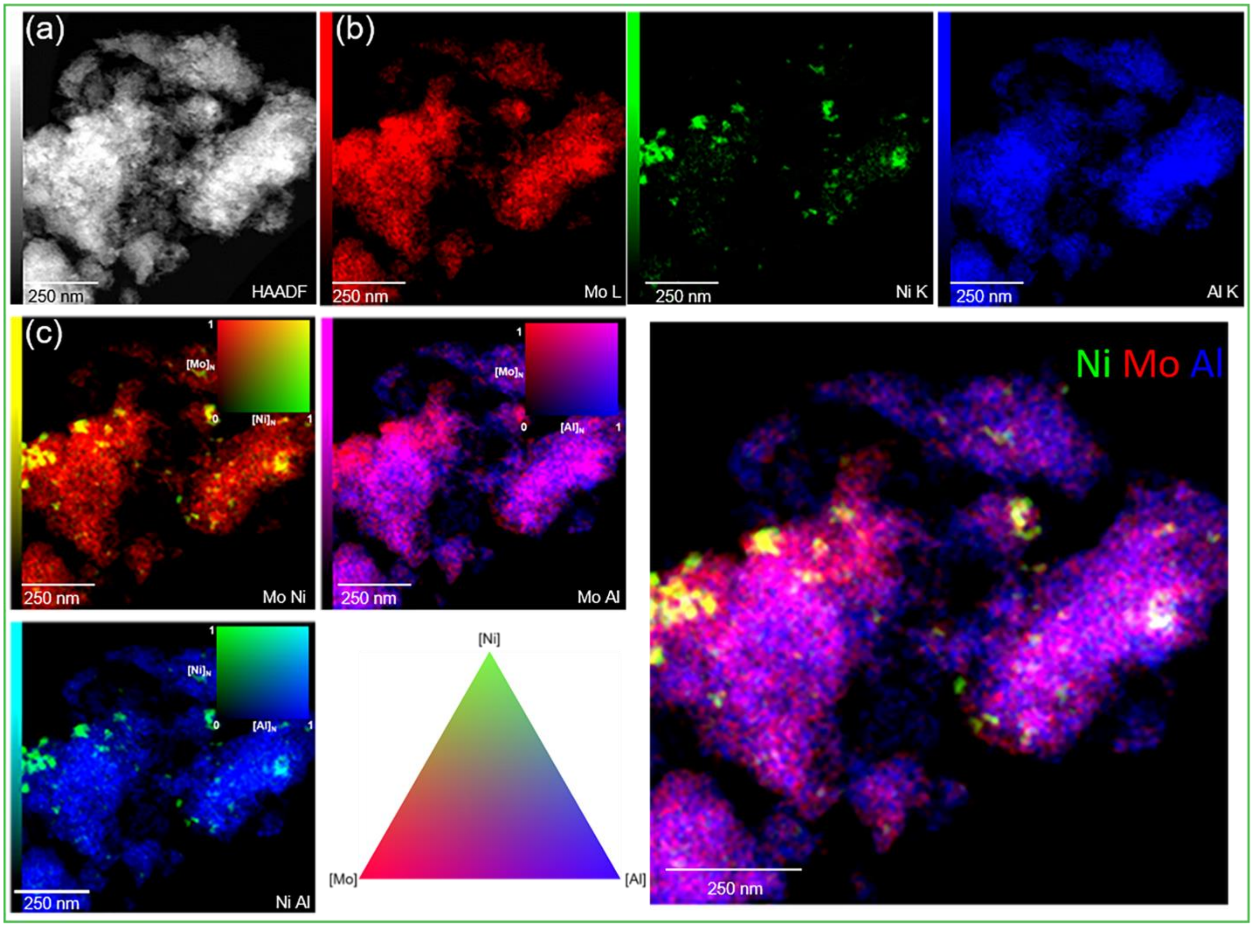


**Figure S7.** Electron microscopy of sample B (spent). a) High-angle annular dark-field (HAADF) STEM image. b) Energy dispersive X-ray spectroscopy (EDX) maps of molybdenum (Mo), nickel (Ni) and aluminum (Al) corresponding to the STEM image of (a). c) Normalized (N) and merged EDX maps of Mo, Ni and Al.

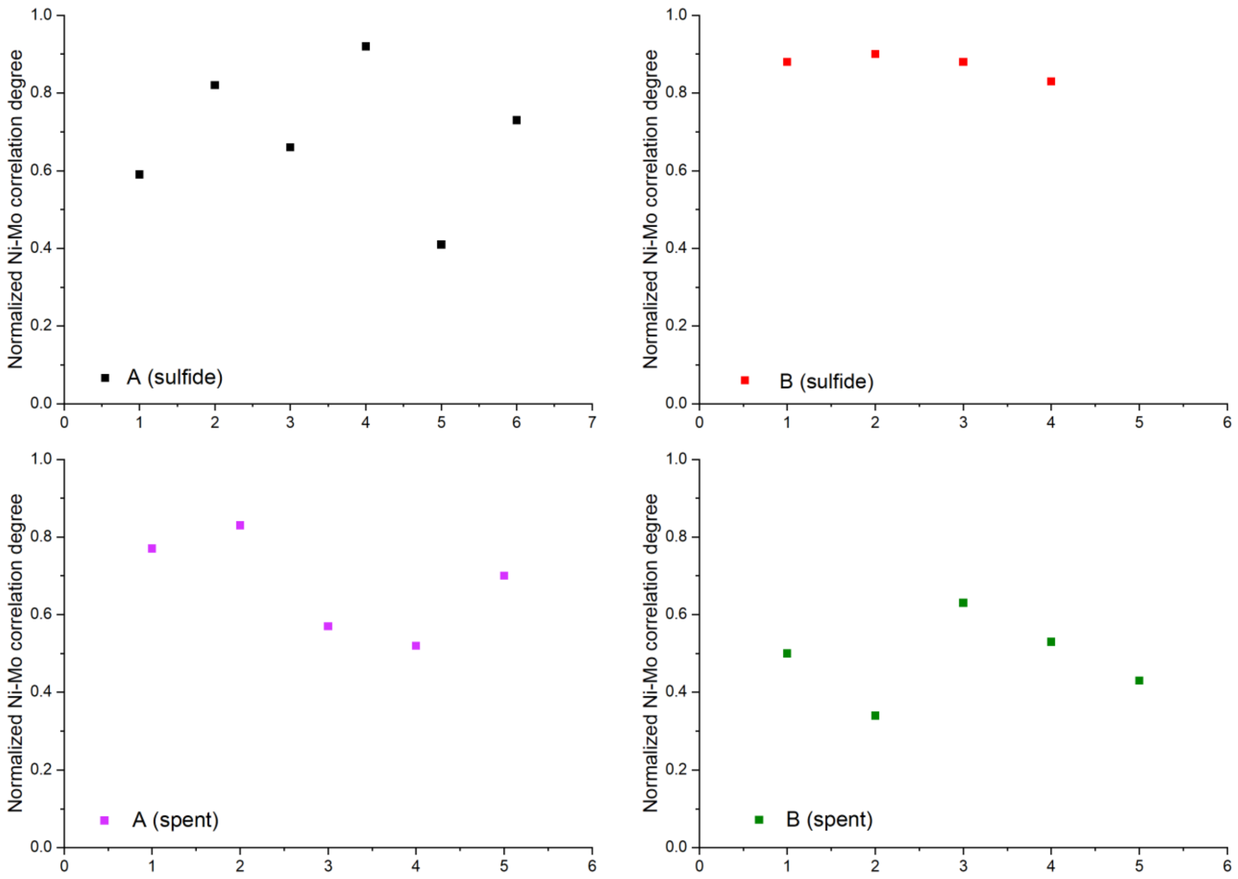


**Figure S8.** Normalized Ni-Mo correlation degree of samples A (sulfide), B (sulfide), A (spent) and B (spent) extracted from EDX mapping data. X-axis is just number of labels for each data point, representing the selected area used for calculation.

**Table S1.** Electron density of the selected reference compounds.

| Compound | Formula | ED $n_e$/Å$^3$ |
|---|---|---|
| Air | | 0.000 |
| water | $H_2O$ | 0.334 |
| Phosphoric acid | $H_3PO_4$ | 0.578 |
| Amorphous Alumina | $Al_2O_3$ | 0.785 |
| Metallic Molybdenum | Mo | 2.689 |
| Metallic Nickel | Ni | 2.561 |
| Molybdenum sulfide | $MoS_2$ | 1.409 |
| Molybdenum trioxide | $MoO_3$ | 1.295 |
| Nickel Oxide | NiO | 1.936 |
| Nickel Sulfide | NiS | 1.795 |
| $NiMO_2$ | 15wt%Mo, 3.67wt%Ni | 1.168 |

The electron densities of considered reference compounds were calculated using tabulated molecular weight and mass density values[17].

**Movie S1-S4.** Volume rendering of ptychographic tomograms. Shown are volume reconstructions of and cut slices through the retrieved electron density tomograms of samples A (sulfide), B (sulfide), A (spent) and B (spent).

**Supporting References:**


1. Holler, M.; Raabe, J.; Wepf, R.; Shahmoradian, S. H.; Diaz, A.; Sarafimov, B.; Lachat, T.; Walther, H.; Vitins, M. OMNY PIN-A versatile sample holder for tomographic measurements at room and cryogenic temperatures. *Rev. Sci. Instrum.* **2017,** *88* (11), 113701.
2. Holler, M.; Ihli, J.; Tsai, E. H. R.; Nudelmann, F.; Verezhak, M.; Berg, W. D. J.; Shahmoradian, S. H. A lathe system for micrometer-sized cylindrical sample preparation at room and cryogenic temperatures. *J. Synchrotron Radiat.* **2020,** *27*, 472-476.
3. Odstrčil, M.; Lebugle, M.; Guizar-Sicairos, M.; David, C.; Holler, M. Towards optimized illumination for high-resolution ptychography. *Opt. Express* **2019,** *27* (10), 14981-14997.
4. Guizar-Sicairos, M.; Johnson, I.; Diaz, A.; Holler, M.; Karvinen, P.; Stadler, H.-C.; Dinapoli, R.; Bunk, O.; Menzel, A. High-throughput ptychography using Eiger: scanning X-ray nano-imaging of extended regions. *Opt. Express* **2014,** *22* (12), 14859-14870.
5. Johnson, I.; Bergamaschi, A.; Billich, H.; Cartier, S.; Dinapoli, R.; Greiffenberg, D.; Guizar-Sicairos, M.; Henrich, B.; Jungmann, J.; Mezza, D. et al. Eiger: a single-photon counting X-ray detector. *J. Instrum.* **2014,** *9* (05), C05032.
6. Ihli, J.; Bloch, L.; Krumeich, F.; Wakonig, K.; Holler, M.; Guizar-Sicairos, M.; Weber, Th.; da Silva, J. C.; van Bokhoven, J. A. Hierarchical structure of NiMo hydrodesulfurization catalysts determined by ptychographic X-Ray computed tomography. *Angew. Chem., Int. Ed.* **2020,** *59* (39), 17266-17271.
7. Holler, M.; Odstrcil, M.; Guizar-Sicairos, M.; Lebugle, M.; Müller, E.; Finizio, S.; Tinti, G.; David, C.; Zusman, J.; Unglaub, W. Three-dimensional imaging of integrated circuits with macro- to nanoscale zoom. *Nat. Electron.* **2019,** *2* (10), 464-470.
8. Holler, M.; Raabe, J. Error motion compensating tracking interferometer for the position measurement of objects with rotational degree of freedom. *Opt. Eng.* **2015,** *54* (5), 054101-054101.
9. Huang, X.; Yan, H.; Harder, R.; Hwu, Y.; Robinson, I. K.; Chu, Y. S. Optimization of overlap uniformness for ptychography. *Opt. Express* **2014,** *22* (10), 12634-12644.
10. Wakonig, K.; Stadler, H.-C.; Odstrčil, M.; Tsai, E. H.; Diaz, A.; Holler, M.; Usov, I.; Raabe, J.; Menzel, A.; Guizar-Sicairos, M. PtychoShelves, a versatile high-level framework for high-performance analysis of ptychographic data. *J. appl. crystallogr.* **2020,** *53* (2), 574-586.
11. Odstrčil, M.; Holler, M.; Raabe, J.; Guizar-Sicairos, M. Alignment methods for nanotomography with deep subpixel accuracy. *Opt. express* **2019,** *27* (25), 36637-36652.
12. van Heel, M.; Schatz, M. Fourier shell correlation threshold criteria. *J. Struct. Biol.* **2005,** *151* (3), 250-262.
13. Howells, M. R.; Beetz, T.; Chapman, H. N.; Cui, C.; Holton, J. M.; Jacobsen, C. J.; Kirz, J.; Lima, E.; Marchesini, S.; Miao, H. et al. An assessment of the resolution limitation due to radiation-damage in X-ray diffraction microscopy. *J. Electron Spectrosc. Relat. Phenom.* **2009,** *170* (1–3), 4-12.
14. Schindelin, J.; Arganda-Carreras, I.; Frise, E.; Kaynig, V.; Longair, M.; Pietzsch, T.; Preibisch, S.; Rueden, C.; Saalfeld, S.; Schmid, B. et al. Fiji: an open-source platform for biological-image analysis. *Nat. Methods* **2012,** *9* (7), 676-682.
15. Groso, A.; Abela, R.; Stampanoni, M. Implementation of a fast method for high resolution phase contrast tomography. *Opt. express* **2006,** *14* (18), 8103-8110.
16. Ihli, J.; Levenstein, M. A.; Kim, Y.-Y.; Wakonig, K.; Ning, Y.; Tatani, A.; Kulak, A. N.; Green, D. C.; Holler, M.; Armes, S. P. et al. Ptychographic X-ray tomography reveals additive zoning in nanocomposite single crystals. *Chem. Sci.* **2020,** *11* (2), 355-363.

17. Diaz, A.; Trtik, P.; Guizar-Sicairos, M.; Menzel, A.; Thibault, P.; Bunk, O., Quantitative x-ray phase nanotomography. *Phys. Rev. B* **2012,** *85* (2), 020104.
